# Targeting Leukopoiesis: Pharmacological and Biotechnological Strategies for the Treatment of Leukopenia

**DOI:** 10.3390/biomedicines14030624

**Published:** 2026-03-11

**Authors:** Lyailya Baktybayeva, Altynay B. Kaldybayeva, Anastassiya Sokolenko, Bagila Tursynova, Assel Yu. Ten, Guldana Daulet, Erkebulan Svambayev, Mario Thevis, Valentina K. Yu, Khaidar S. Tassibekov

**Affiliations:** 1 Laboratory of Chemistry of Synthetic and Natural Medicinal Compounds, A.B. Bekturov Institute of Chemical Sciences, Almaty 050010, Kazakhstan; lilaturap707@gmail.com (L.B.); tursynova_0101@mail.ru (B.T.); ten-assel@mail.ru (A.Y.T.); yuvkconst@gmail.com (V.K.Y.); kh.tassibekov@ihn.kz (K.S.T.); 2 Department of Biophysics, Biomedicine and Neuroscience, Al-Farabi Kazakh National University, Almaty 050040, Kazakhstan; daulet.guldana2021@gmail.com (G.D.); erkebulan.svambayev@gmail.com (E.S.); 3 Department of Chemistry and Technology of Organic Substances, Natural Compounds and Polymers, Al-Farabi Kazakh National University, Almaty 050040, Kazakhstan; 4 Faculty of Natural Sciences and Geography, Abai Kazakh National Pedagogical University, Almaty 050010, Kazakhstan; 5 Center for Preventive Research on Doping, Institute of Biochemistry, German Sports University, 50933 Cologne, Germany; thevis@dshs-koeln.de

**Keywords:** leukopoiesis-stimulating drugs, drugs of bone marrow origin, drugs of microbial origin, drugs of thymic origin, drugs of cytokine origin, drugs of plant origin, drugs of chemically pure origin, imidazole-containing drugs

## Abstract

Leukopenia remains a major clinical challenge associated with infectious diseases, oncological therapies, autoimmune disorders, and metabolic and iatrogenic conditions. Insufficient leukopoiesis not only increases susceptibility to infections but also limits the intensity and continuity of anticancer and immunosuppressive treatments. Targeted stimulation of leukopoiesis therefore represents a critical therapeutic strategy in modern biomedicine. This narrative review summarizes pharmacological and biotechnological approaches to leukopoiesis stimulation based on an analysis of peer-reviewed literature from major biomedical databases. Emphasis was placed on molecular mechanisms of action, clinical positioning, and translational potential of leukopoiesis-modulating agents. Current leukopoiesis-stimulating strategies encompass cytokine-based therapies, bone marrow-derived peptides, thymic and microbial immunomodulators, nucleic acid-based agents, plant-derived compounds, and chemically synthesized small molecules. Classical colony-stimulating factors remain the cornerstone of clinical practice; however, their limitations, including adverse effects and restricted spectrum of action, have driven the development of alternative approaches. Emerging strategies increasingly target specific regulatory nodes of hematopoiesis, including bone marrow stromal interactions, transcription factor signaling, chemokine receptor pathways, and immune cell differentiation programs. Advances in the understanding of leukopoiesis regulation have expanded therapeutic opportunities beyond conventional growth factor administration. Pharmacological and biotechnological targeting of leukopoiesis holds promise for improving clinical outcomes in patients with leukopenia of diverse etiologies. Future progress in this field will depend on the integration of mechanistic insights with clinical evidence to enable more selective, effective, and safer leukopoiesis-stimulating therapies.

## 1. Introduction

Leukopoiesis is a tightly regulated biological process responsible for the continuous generation of leukocytes that sustain innate and adaptive immune responses. Disruption of leukopoiesis leads to leukopenia, a clinically significant condition characterized by an increased susceptibility to infections, impaired immune surveillance, and reduced tolerance to pharmacological interventions. Leukopenia frequently develops in the context of oncological chemotherapy, radiotherapy, severe infections, autoimmune disorders, metabolic diseases, organ transplantation, and prolonged exposure to cytotoxic or immunosuppressive agents. In these settings, insufficient leukocyte recovery represents not only a biological consequence of disease or treatment but also a major limiting factor for effective clinical management.

Despite substantial advances in supportive care, leukopenia remains a persistent and unresolved challenge in modern medicine. In oncological practice, leukocyte depletion often necessitates dose reductions or delays in chemotherapy, thereby compromising therapeutic efficacy. In infectious and inflammatory diseases, impaired leukopoiesis contributes to recurrent or severe infections, prolonged hospitalization, and increased mortality. These clinical realities underscore the critical need for therapeutic strategies that restore or enhance leukocyte production in a controlled and physiologically relevant manner [1,2].

At present, pharmacological stimulation of leukopoiesis relies predominantly on colony-stimulating factors (CSFs), particularly granulocyte colony-stimulating factor (G-CSF) and granulocyte–macrophage colony-stimulating factor (GM-CSF). While these agents have transformed the management of chemotherapy-induced neutropenia and bone marrow recovery, their clinical use is associated with several limitations. These include adverse effects, limited efficacy in certain patient populations, narrow lineage specificity, and the inability to fully recapitulate the complex regulatory environment of the bone marrow niche. Moreover, CSF-based therapies primarily address terminal stages of leukocyte differentiation, leaving upstream regulatory mechanisms largely untargeted.

Growing insights into the molecular and cellular regulation of hematopoiesis have revealed that leukopoiesis is controlled by a highly interconnected network involving bone marrow stromal cells, cytokines, transcription factors, chemokine gradients, metabolic cues, and immune-mediated feedback mechanisms. This expanded understanding has catalyzed interest in alternative leukopoiesis-stimulating strategies that extend beyond classical growth factors. These approaches aim to modulate leukocyte production at multiple regulatory levels, including stem and progenitor cell maintenance, lineage commitment, differentiation, and functional maturation [3].

In recent years, diverse pharmacological and biotechnological agents with leukopoiesis-modulating properties have been investigated. These include bone marrow-derived regulatory peptides, thymic peptides, muramyl dipeptide derivatives, nucleic acid-based agents, plant-derived immunomodulators, and chemically synthesized small molecules. In parallel, advances in biotechnology have enabled the development of targeted biologics, nanoparticle-based systems, and receptor-specific modulators that influence leukopoiesis through defined molecular targets. Such strategies hold promise for achieving more selective, durable, and safer stimulation of leukocyte production compared with conventional therapies [4].

The increasing clinical heterogeneity of leukopenic conditions further reinforces the need for diversified therapeutic options. Leukopenia arising from cytotoxic therapy differs fundamentally from leukopenia associated with chronic inflammation, metabolic dysregulation, or immune exhaustion, suggesting that a “one-size-fits-all” approach is unlikely to be effective. Consequently, there is growing interest in tailoring leukopoiesis-stimulating interventions to specific pathological contexts and underlying regulatory mechanisms.

In this review, we summarize current pharmacological and biotechnological strategies aimed at targeting leukopoiesis for the treatment of leukopenia. Emphasis is placed on molecular mechanisms of action, clinical applications, and emerging therapeutic concepts that extend beyond traditional growth factor administration. By integrating mechanistic insights with clinical evidence, this review highlights both established and developing approaches that may shape the future landscape of leukopoiesis-stimulating therapies.

## 2. Methods

### 2.1. Literature Search Strategy

This narrative review was conducted in accordance with general recommendations for non-systematic reviews published in biomedical journals. A comprehensive literature search was performed using the PubMed/MEDLINE, Scopus, and Web of Science databases. The search strategy combined Medical Subject Headings (MeSH) terms and free-text keywords related to leukopoiesis and leukopenia, including but not limited to: “leukopoiesis”, “leukopenia”, “neutropenia”, “granulopoiesis”, “colony-stimulating factor”, “G-CSF”, “GM-CSF”, “hematopoietic regulation”, “bone marrow niche”, “immunomodulators”, “thymic peptides”, “muramyl dipeptide”, “CXCR4 antagonist”, “transcription factors”, and “leukocyte differentiation”.

### 2.2. Inclusion and Exclusion Criteria

Peer-reviewed articles published in English were considered eligible for inclusion. The primary focus was placed on studies describing molecular mechanisms, pharmacological properties, biological activity, clinical applications, and safety profiles of leukopoiesis-stimulating agents. Both experimental and clinical studies were included if they provided mechanistic or translational insight relevant to leukocyte production or recovery.

Exclusion criteria comprised non-peer-reviewed sources, abstracts without full-text availability, and studies lacking clear relevance to leukopoiesis or leukopenia. Reports exclusively addressing erythropoiesis or thrombopoiesis without implications for leukocyte regulation were also excluded.

### 2.3. Time Frame and Study Selection

The primary time frame of the literature search covered publications from 2000 to 2025, reflecting the period of rapid advancement in immunology, hematopoiesis, and biotechnological drug development. Seminal earlier studies were selectively included when necessary to illustrate the historical development of leukopoiesis-stimulating therapies or to contextualize modern approaches.

The final selection of publications was based on relevance, scientific quality, and contribution to understanding leukopoiesis regulation, therapeutic efficacy, or clinical positioning.

The initial database search generated approximately 320 records. Following the removal of duplicates and a screening process based on title and abstract relevance, over 200 publications were chosen for full-text analysis. The final selection was determined through a qualitative assessment of the methodological rigour, relevance to leukopoiesis regulation, and potential contribution to translational and clinical understanding. The studies were grouped thematically based on molecular targets, level of hematopoietic regulation, and clinical relevance. Given the nature of this narrative review, a formal PRISMA flow chart was not created; however, the selection process was guided by principles of transparency and relevance.

### 2.4. Data Analysis and Synthesis

The selected literature was analyzed qualitatively and synthesized into thematic sections corresponding to major pharmacological and biotechnological classes of leukopoiesis-stimulating agents. Emphasis was placed on identifying shared and distinct mechanisms of action, clinical indications, limitations of existing therapies, and emerging molecular targets. No formal meta-analysis was performed, as the scope of this review encompassed heterogeneous therapeutic strategies and study designs.

## 3. Biological Regulation of Leukopoiesis: Key Molecular and Cellular Targets

Leukopenia can arise from various pathological mechanisms, which can be broadly classified into three main categories: (1) reduced production due to impaired or suppressed bone marrow function; (2) enhanced peripheral destruction or depletion; and (3) abnormal distribution or sequestration. Reduced production may result from cytotoxic treatment, aplastic anemia, marrow infiltration, nutritional deficiencies (e.g., vitamin B12 or folate deficiency), or congenital stem cell abnormalities. Enhanced destruction may occur in cases of autoimmune neutropenia, hypersplenism, or drug-induced immunological reactions. Redistribution is seen in severe infections and inflammatory conditions. Understanding these mechanisms is crucial for the rational selection of strategies to stimulate leukopoiesis, as treatments targeting stem cell proliferation may not be effective in conditions primarily caused by peripheral depletion. Leukopenia may occur due to decreased production of leukocytes caused by suppression of the bone marrow, increased peripheral destruction, or abnormal sequestration of cells. Infections, chemotherapy, autoimmune phenomena, hematological malignancies, and nutritional deficiencies are major contributing factors to its development, with each having distinct pathophysiological effects [5].

Leukopoiesis is the tightly controlled process by which hematopoietic stem and progenitor cells differentiate into mature leukocytes. Its regulation depends on the integration of cellular components, soluble mediators, and transcriptional networks, making it a multilevel biological process that ensures balanced immune cell production in steady state and during stress [6,7]. The bone marrow niche, bone marrow mesenchymal stem/stromal cells (BM-MSCs), cytokines and colony-stimulating factors (CSFs), PU.1 and GATA-1/GATA-2 and stromal factors represent key cellular and molecular targets in the biological regulation of leukopoiesis.

The bone marrow niche provides a highly specialized microenvironment that governs leukopoiesis by integrating structural, molecular, and cellular cues essential for hematopoietic stem and progenitor cell (HSPC) maintenance and differentiation. Rather than serving as a passive scaffold, the niche actively regulates HSPC quiescence, self-renewal, lineage commitment, and mobilization through dynamic interactions between hematopoietic cells and surrounding stromal components [8,9]. Spatially and functionally distinct niches, including endosteal and perivascular compartments, contribute to the fine-tuning of leukocyte production under both steady-state and stress conditions [10].

BM-MSCs are central cellular components of the bone marrow niche and play a critical role in leukopoiesis by indirectly regulating hematopoietic stem and progenitor cells. These multipotent stromal cells secrete key regulatory factors such as stem cell factor (SCF) and CXCL12, which promote progenitor cell survival, retention, and controlled differentiation. In addition, BM-MSCs contribute to immune modulation and inflammatory signaling, thereby influencing myeloid lineage commitment and leukocyte output during both homeostasis and pathological conditions [11].

Cytokines and colony-stimulating factors are essential soluble regulators of leukopoiesis that control progenitor cell proliferation, survival, and lineage specification. CSFs such as granulocyte colony-stimulating factor (G-CSF), macrophage colony-stimulating factor (M-CSF), and granulocyte–macrophage colony-stimulating factor (GM-CSF) selectively drive the expansion and differentiation of granulocytic and monocytic lineages. Interleukins, including IL-3 and IL-6, act on early hematopoietic progenitors to enhance responsiveness to lineage-specific signals. The precise concentration and temporal availability of these factors determine leukocyte production under physiological and stress-induced conditions [12].

Lineage commitment during leukopoiesis is critically governed by transcription factors, notably PU.1 and the GATA family members GATA-1 and GATA-2. PU.1 is indispensable for myeloid and lymphoid differentiation, with elevated expression favoring granulocyte and macrophage development. GATA-1 primarily regulates erythroid and megakaryocytic differentiation but indirectly influences leukopoiesis through antagonistic interactions with PU.1. GATA-2 is highly expressed in early hematopoietic stem and progenitor cells, where it maintains stemness and proliferative capacity. The dynamic balance and cross-regulation between PU.1 and GATA factors are key determinants of leukocyte lineage fate [13].

Stromal factors bridge the gap between the cellular niche and molecular signaling pathways. Produced by stromal cells, these factors regulate progenitor cell localization, adhesion, survival, and responsiveness to cytokines. By shaping the microenvironment and fine-tuning signal availability, stromal factors coordinate spatial and temporal aspects of leukopoiesis. They therefore represent microenvironmental molecular targets essential for controlled leukocyte production [14].

Based on these regulatory mechanisms, current pharmacological and biotechnological strategies targeting leukopoiesis can be classified according to their molecular targets, level of action, and clinical positioning (Table 1).

## 4. Pharmacological and Biotechnological Strategies Targeting Leukopoiesis

Pharmacological and biotechnological approaches targeting leukopoiesis differ substantially in their mechanisms of action, level of hematopoietic regulation, and clinical applicability. The therapeutic strategies discussed in this review differ substantially in their level of clinical validation, ranging from well-established clinical interventions to preclinical and emerging experimental approaches (Table 1). Detailed information is available in the Supplementary Materials (Table S1).

In this paper, “leukopoiesis stimulation” specifically refers to the direct enhancement of the proliferation and differentiation of hematopoietic progenitors within the bone marrow. By contrast, “immune activation” and “immunomodulation” refer to indirect regulation of leukocyte function or cytokine networks, which may secondarily influence leukocyte counts [15]. These distinctions have been maintained throughout the paper in order to preserve conceptual accuracy.

For clarity, the therapeutic strategies discussed below are presented in order of their clinical maturity: 1. Globally established and guideline-supported therapies, such as colony-stimulating factors. 2. Regionally approved or selectively used immunomodulators. 3. Emerging or preclinical experimental platforms. This stratification is intended to help readers distinguish clinically proven interventions from investigational approaches. The GRADE systems were used to assess the quality of evidence and strength of recommendations in the review [16].

To ensure conceptual clarity, the therapeutic approaches discussed below are interpreted within a hierarchy of evidence that is explicitly defined. Clinically established agents that have regulatory approval and have undergone large-scale clinical trials are distinguished from therapies that have been approved by regional authorities and are supported by limited or heterogeneous clinical trial data, as well as from preclinical and early experimental strategies that are primarily supported by in vitro and animal studies.

### 4.1. Bone Marrow-Targeted Approaches

#### 4.1.1. Bone Marrow-Derived Regulatory Peptides and Stromal Cell-Mediated Signaling

Bone marrow mesenchymal stem cells (BM-MSCs) are an essential component of the hematopoietic microenvironment. While they represent a small fraction of the total nucleated cells in the bone marrow, they exhibit the ability to differentiate into multiple lineages and exert regulatory effects on hematopoietic processes through direct cell–cell interactions and secreted factors [17,18]. In addition to their differentiation potential, BM-MSCs primarily function as regulatory stromal cells that secrete a diverse range of cytokines, chemokines, growth factors, and other stromal factors involved in maintaining hematopoiesis and modulating immune responses.

Within the bone marrow microenvironment, bone marrow-derived mesenchymal stem cells (BM-MSCs) support the proliferation of hematopoietic stem cells (HSCs) and influence their lineage commitment, including the differentiation of leukocytes [19,20,21,22]. Numerous studies have shown that MSCs, when introduced into damaged tissues or organs, are able to restore tissue structure and function. MSCs can be derived not only from the bone marrow, but also from other sources such as umbilical cord blood, subcutaneous adipose tissue, and the placenta [23,24]. The immunomodulatory effects of MSCs include suppression of the activation of excessive T cells, NK cells, and dendritic cells, promotion of the expansion of regulatory T cells, and modulation of cytokine networks through the secretion of soluble mediators such as transforming growth factor-β (TGF-β), interleukin-10 (IL-10), prostaglandin E2 (PGE2), indoleamine 2,3-dioxygenase (IDO), and hepatocyte growth factor (HGF) [25,26,27,28,29,30]. In addition, factors derived from MSCs contribute to anti-apoptotic signaling and provide vascular support under stress conditions, indirectly facilitating hematopoietic recovery.

Despite these pleiotropic effects, the clinical translation of mesenchymal stem cell (MSC)-based therapies remains a challenging task. Intravenously administered MSCs often undergo pulmonary sequestration or complement-mediated clearance, and inter-donor variability, as well as the complexity of manufacturing processes, contribute to inconsistent therapeutic outcomes. These limitations have prompted interest in more targeted regulatory strategies [31,32,33,34].

Recent efforts focus on transcriptional regulation of hematopoiesis, specifically the modulation of PU.1 and GATA transcription factors, which govern myeloid-erythroid lineage decisions. Delivery systems based on nanoparticles aim to influence these transcriptional networks in order to enable more precise control over early hematopoietic differentiation pathways. Theoretically, such approaches may allow for lineage prioritization without the need for supraphysiological stimulation from colony-stimulating factors [35,36,37,38].

However, both MSC-derived peptide approaches and nanoparticle-mediated transcription factor targeting remain largely preclinical. Current evidence derives predominantly from in vitro and animal studies, while major translational barriers—including biodistribution control, long-term safety, scalability, and regulatory complexity—remain unresolved [39]. Therefore, these strategies should be regarded as investigational platforms with potential complementary roles rather than clinically validated alternatives to established CSF therapy [40,41].

#### 4.1.2. Myelopeptides as Endogenous Regulators of Leukopoiesis

Myelopeptides are low-molecular-weight regulatory peptides of bone marrow origin that participate in endogenous control of hematopoiesis and immune function. Both natural and synthetic myelopeptide-based drugs have been described. Initial studies identified short peptide sequences synthesized by bone marrow cells, followed by determination of their amino acid composition and biological activity [42]. A set of primary myelopeptides capable of modulating leukocyte proliferation, differentiation, and immune responsiveness is summarized in Table 2.

Unlike exogenous hematopoietic stimulators such as colony-stimulating factors (CSFs), myelopeptides function as endogenous bioregulatory mediators, acting through ligand–receptor interactions to fine-tune immune cell activation and differentiation. Their activity reflects physiological signaling within the bone marrow microenvironment rather than supraphysiological stimulation.

**Figure 1:**
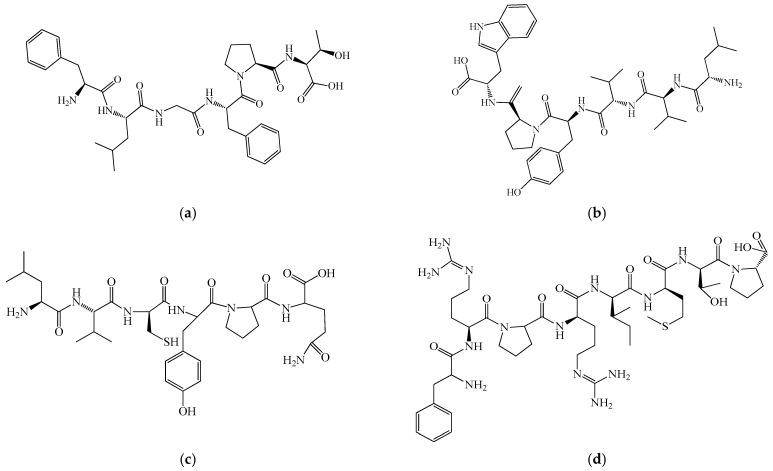
Primary structure of immunoregulatory and monocytic-modulating myelopeptides: (**a**) MP-1; (**b**) MP-2; (**c**) MP-3; (**d**) MP-4. Short linear amino acid sequences highlight proline-rich and aromatic residues potentially involved in receptor-mediated signaling.

**Figure 2:**
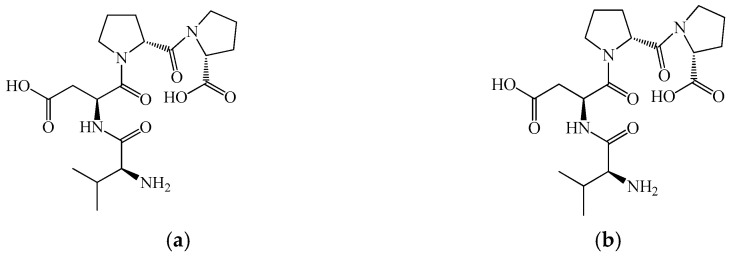
Primary structure of differentiation-inducing myelopeptides: (**a**) MP-5; (**b**) MP-6. These short peptides are associated with regulation of terminal myeloid and erythroid differentiation.

As shown in Figure 1 and Figure 2, primary myelopeptides are short linear peptides ranging from four to eight amino acids. Despite their minimal length, they contain conserved structural motifs, including proline-rich segments and hydrophobic residues, which are characteristic of regulatory peptides involved in receptor-mediated signaling. The limited size of these molecules likely facilitates rapid diffusion within the bone marrow microenvironment and transient, context-dependent receptor interactions.

Functionally, myelopeptides can be grouped into immunoregulatory, differentiation-inducing, and antiproliferative factors. MP-1 (Figure 1a) and MP-2 (Figure 1b) primarily exert immunoregulatory effects by restoring functional activity of T- and B-lymphocytes under conditions of immune suppression. MP-1 interacts with CD4^+^ T lymphocytes, leading to normalization of antibody production and restoration of the CD4^+^/CD8^+^ balance characteristic of immunodeficiency states [42]. The short linear structure of MP-1 reflects its capacity for receptor-mediated signaling rather than enzymatic or structural activity, consistent with its role as a regulatory peptide. MP-2 counteracts tumor-induced immune suppression by restoring CD4 receptor expression and functional competence of T cells inhibited by leukemic cell-derived factors [43,44]. Both peptides enhance proliferative activity of immune effector cells and activate innate immune mechanisms without inducing excessive or uncontrolled stimulation [45].

Notably, MP-1 and MP-2 contain aromatic residues (Phe, Tyr, Trp) and proline motifs that may contribute to conformational rigidity and receptor affinity, potentially underlying their immunoregulatory activity. In contrast, differentiation-inducing peptides such as MP-4, MP-5, and MP-6 exhibit shorter or arginine-containing sequences, which may influence their interaction with membrane-associated regulatory complexes involved in lineage commitment. MP-3 (Figure 1c) predominantly modulates innate immune responses, enhancing phagocytic and antigen-presenting functions of myeloid cells [46,47,48,49,50]. The presence of cysteine in MP-3 may be functionally relevant, as thiol-containing residues can participate in redox-sensitive signaling pathways or stabilize peptide conformation, potentially contributing to its modulatory effects on monocytic cells. In contrast, MP-4 (Figure 1d), MP-5 (Figure 2a), and MP-6 (Figure 2b) act as differentiation factors, promoting terminal differentiation of leukemic myeloid and erythroid cell lines and relieving differentiation blocks characteristic of malignant hematopoiesis. These peptides therefore appear to participate in endogenous regulation of lineage commitment rather than direct mitogenic stimulation [51].

Based on mixtures of myelopeptides, the drug Myelopid has been developed and is used in veterinary medicine for prevention and treatment of acquired immunodeficiency states, further supporting the regulatory rather than stimulatory nature of these peptides [52].

Interestingly, myelopeptides also exhibit opioid-like activity and can interact with opioid receptors of the nervous system, producing mild analgesic effects [53]. This observation provides a functional link between neuroendocrine and immune regulation. In contrast to chronic or high-dose exogenous opioid administration—which suppresses hematopoiesis and immune cell proliferation—endogenously produced bone marrow peptides may contribute to balanced immune modulation [54,55,56,57,58,59,60,61].

The structural simplicity illustrated in Figure 1 and Figure 2 underscores a key feature of myelopeptides: their biological activity appears to depend less on complex tertiary structure and more on short functional motifs capable of modulating receptor-mediated signaling. This distinguishes them from large cytokines and growth factors and supports their classification as endogenous microregulatory peptides.

Collectively, these findings support the concept that myelopeptides represent an intrinsic regulatory layer of hematopoiesis and immunity. Unlike exogenous cytokines or CSFs, which induce strong and often unidirectional stimulation, myelopeptides provide context-dependent, homeostatic regulation of immune cell proliferation, differentiation, and function. However, it should be emphasized that the clinical validation of myelopeptides remains limited. Most available data originate from experimental studies, small-scale clinical observations, or veterinary applications, with a lack of large randomized controlled trials meeting contemporary evidence-based medicine standards. Consequently, although myelopeptides represent biologically intriguing endogenous regulators, their therapeutic positioning in human leukopenia or immune deficiency remains investigational rather than established [62,63].

### 4.2. Cytokine-Based Therapies

#### 4.2.1. Colony-Stimulating Factors in Clinical Practice

Colony-stimulating factors remain the cornerstone of clinical leukopoiesis stimulation, acting primarily at late stages of granulocytic differentiation (Table 1). Drugs based on colony-stimulating factors (CSFs) are widely used in clinical practice. Their major applications include hematopoietic stem cell mobilization for transplantation, activation of donor bone marrow prior to stem cell collection, prevention and treatment of febrile neutropenia during chemotherapy, and stimulation of hematopoiesis in patients with severe secondary immunosuppressive conditions.

Within national and international bone marrow donation programs, including “Be the Match”, donors routinely receive filgrastim injections for several days prior to stem cell collection. Filgrastim stimulates the release of hematopoietic stem and progenitor cells from the bone marrow into the peripheral blood. The most commonly used CSF-based drugs in transplantation are filgrastim, lenograstim, and pegfilgrastim. Filgrastim and lenograstim are granulocyte colony-stimulating factors (G-CSF), whereas pegfilgrastim is a pegylated form with an extended half-life and prolonged biological activity. Additional hematopoietic growth factors include ancestim (stem cell factor) and sargramostim, a granulocyte–macrophage colony-stimulating factor (GM-CSF) [64,65,66]. Both G-CSF and GM-CSF are used clinically to enhance leukocyte recovery.

A major therapeutic indication for G-CSF is its use during cytotoxic chemotherapy to prevent or treat febrile neutropenia. Administration of G-CSF allows maintenance or escalation of chemotherapy dose intensity and is mandatory in patients with a history of neutropenic fever. G-CSF is also administered as part of the standard treatment once febrile neutropenia has developed [67,68,69,70,71,72,73].

Myelosuppression remains a frequent complication of chemotherapy, manifesting as neutropenia, anemia, thrombocytopenia, and subsequent infectious complications in 25–40% of previously untreated patients. Bone marrow hypoplasia typically reaches its nadir 1–2 weeks after chemotherapy, with severity influenced by drug regimen, cumulative dose, prior radiation therapy, comorbidities, and overall patient condition. Febrile neutropenia, defined as a body temperature above 38.3 °C lasting longer than one hour in the absence of an identifiable cause, represents a medical emergency. Because clinical signs of infection are often absent and microbiological confirmation may take several days, prompt initiation of CSF-based therapy is critical and frequently life-saving [74].

Currently approved CSF drugs include filgrastim (Neupogen), pegfilgrastim (Neulasta), sargramostim (Leukine), and the newer recombinant G-CSF tbo-filgrastim (Neutroval), which has received FDA approval. Tbo-filgrastim is a short-acting G-CSF used for the treatment and prevention of febrile neutropenia in cancer patients receiving platinum-based chemotherapy and is marketed in Europe as Tevagrastim [75,76,77,78,79,80]. GM-CSF-based drugs are also employed in combination with stem cell therapies, salvage regimens for metastatic cancer, and in selected infectious and inflammatory conditions [81,82]. Ongoing research continues to explore novel CSF-based and cytokine-modulating drugs for oncological, fungal, and bacterial diseases [83,84,85,86].

Despite their proven clinical efficacy, CSF-based therapies have several important limitations. First, CSFs induce strong but lineage-restricted stimulation, primarily affecting granulocytic or myelomonocytic compartments, with limited influence on early hematopoietic decision-making or balanced lineage differentiation. Second, CSF administration is associated with adverse effects, including bone pain, fever, splenomegaly, thrombotic complications, and exacerbation of inflammatory responses, particularly with repeated or high-dose use. Rare but serious complications such as splenic rupture and acute respiratory distress have also been reported.

Third, reduced responsiveness or functional resistance to CSF therapy can occur in heavily pretreated patients, in advanced malignancies, or in the context of chronic bone marrow failure, limiting long-term effectiveness. Finally, CSF-based treatments impose a substantial economic burden due to repeated dosing requirements, high manufacturing costs of recombinant proteins, and the need for close clinical monitoring. Together, these limitations highlight the need for complementary or alternative approaches that more closely reflect endogenous regulation of hematopoiesis rather than exogenous supraphysiological stimulation [87].

#### 4.2.2. Interleukins and Cytokine Complexes

Interleukins (ILs), a term introduced by Vern Paetkau in 1979, represent a large family of cytokines originally identified as mediators of communication between leukocytes. Interleukins regulate cell growth, differentiation, survival, and functional activation within the immune system and play a central role in coordinating innate and adaptive immune responses [88,89].

In clinical practice, most interleukin-targeting drugs are used not to stimulate hematopoiesis but to suppress excessive or pathological immune activation. These include inhibitors of TNF-α (etanercept, infliximab, adalimumab), IL-1 signaling (anakinra, rilonacept, canakinumab, endogenous IL-1RA), IL-2 receptor signaling (daclizumab, basiliximab), IL-5 (mepolizumab), IL-6 (tocilizumab, siltuximab), IL-4/IL-13 pathways (pitrakinra, tralokinumab, lebrikizumab), as well as broader cytokine or JAK–STAT pathway inhibitors (ruxolitinib, ustekinumab, vidofludimus). Several of these agents are widely used in asthma, autoimmune diseases, and chronic inflammatory conditions. In contrast, only a limited number of interleukin-based drugs exhibit immune-stimulating activity. Aldesleukin (recombinant human IL-2) promotes proliferation and activation of IL-2-dependent T lymphocytes, NK cells, and lymphokine-activated killer cells, enhances interferon-γ production, and increases cytotoxic activity. Clinically, aldesleukin is used in selected oncological indications, including metastatic melanoma and renal cell carcinoma [90,91,92,93].

Complex cytokine preparations such as leukinferon and superlymph contain mixtures of endogenous cytokines produced in vitro under immune stimulation. These preparations primarily modulate innate immune responses, enhancing phagocyte migration, phagocytosis, antimicrobial activity, antigen presentation, and local tissue regeneration. Their effects are largely immunoregulatory and localized rather than systemic stimulators of hematopoiesis.

Roncoleukin, a recombinant IL-2 preparation, functions as a central immune regulator by activating proliferation and differentiation of T cells, B cells, and NK cells expressing the CD25 receptor. Its biological effects include enhanced cytotoxicity, immunoglobulin production, antigen presentation, and activation of monocytes and eosinophils. Betaleukin, a recombinant IL-1β preparation, plays a key role in activating innate immunity and early inflammatory responses [94].

Despite their profound immunomodulatory activity, interleukins are not considered direct stimulators of leukopoiesis in the same sense as colony-stimulating factors (CSFs). CSFs such as G-CSF and GM-CSF act directly on hematopoietic progenitor cells, driving their proliferation, survival, and lineage-specific differentiation within the bone marrow. In contrast, most interleukins exert their effects indirectly, primarily by regulating immune cell activation, cytokine networks, and inflammatory signaling.

Interleukins influence leukocyte numbers mainly through secondary mechanisms, including induction of endogenous CSF production, modulation of stromal and immune cell interactions, and alteration of the bone marrow microenvironment. For example, IL-1β enhances leukopoiesis not by directly stimulating progenitor proliferation, but by inducing the synthesis of CSFs and other hematopoietic cytokines by stromal cells, macrophages, and endothelial cells. Similarly, IL-2 primarily expands activated lymphocyte populations rather than promoting de novo leukocyte generation from hematopoietic stem cells.

Thus, interleukins function as immune system regulators rather than primary hematopoietic growth factors, shaping the intensity, quality, and coordination of immune responses. Their role in leukopoiesis is context-dependent and mediated through immune–stromal crosstalk, distinguishing endogenous immune regulation from exogenous, lineage-restricted stimulation provided by CSF therapy.

### 4.3. Microbial- and Nucleic Acid-Derived Immunomodulators

#### 4.3.1. Muramyl Dipeptide Derivatives

Currently, several dozen muramyl dipeptide (MDP) derivatives have been synthesized. Some of these compounds have undergone clinical trials, while others have already been introduced into clinical practice. Native MDP exhibits pronounced adjuvant activity and the ability to stimulate clonal expansion of immunocompetent cells. MDP derivatives enhance antitumor immunity by activating subpopulations of T lymphocytes, natural killer (NK) cells, and by inducing the production of multiple cytokines. However, native MDP is characterized by high pyrogenicity and a range of systemic side effects, which ultimately precluded its widespread clinical use. This limitation stimulated the development of structural analogs with reduced pyrogenicity and enhanced immunostimulatory activity. In particular, the conjugation of MDP with fatty acids or phospholipids resulted in the generation of more lipophilic compounds with altered pharmacokinetic and biological properties [95,96].

Among the lipophilic MDP derivatives currently evaluated in clinical trials is N-acetylglucosamine–N-acetylmuramyl-L-alanyl-D-isoglutamine-L-alanyl-glyceryl dipalmitate (trade name ImmTher). This compound has undergone phase I clinical trials in patients with inoperable tumors and liver metastases. Repeated injections induced leukocytosis, predominantly due to neutrophils, and significantly increased tumor necrosis factor (TNF) levels. A positive clinical response was observed in 3 of 12 patients.

Another extensively studied derivative is MTP-PE, in which dipalmitoylphosphatidylethanolamine is covalently linked to a muramyl tripeptide and incorporated into liposomes (MTP-PE/MLV). This formulation exhibits a strong capacity to activate the tumoricidal activity of macrophages both in vivo and in vitro. Clinical trials were conducted in patients with inoperable tumors and liver metastases (phase I), as well as in patients with melanoma and osteosarcoma (phase II). In these studies, treatment with MTP-PE was associated with a significant increase in overall survival compared with control groups [97].

MDP is known to act synergistically with several cytokines and inflammatory mediators. In combination with TNF, MDP enhances IL-6 secretion by human monocytes, while together with IL-2 or IL-4 it promotes the proliferation and differentiation of B lymphocytes. Platelet-activating factor and MDP act synergistically to induce IL-1 and TNF production by monocyte–macrophages. In addition, the MDP derivative muroctosin, as well as MDP in combination with interferon-γ, more potently induce cytokine synthesis and tumoricidal activity in both human and murine macrophages.

MDP derivatives are of considerable mechanistic interest because they represent minimal structural motifs of bacterial peptidoglycan capable of activating innate immune responses through the intracellular pattern-recognition receptor NOD2. Engagement of NOD2 leads to NF-κB and MAPK signaling, resulting in the production of pro-inflammatory cytokines, chemokines, and colony-stimulating factors, thereby linking innate and adaptive immunity. However, despite their potent immunostimulatory activity, the clinical application of MDP derivatives has been constrained by systemic toxicity, including pyrogenicity, excessive cytokine release, and limited therapeutic windows. Furthermore, variability in NOD2 expression and signaling efficiency among patients, as well as difficulties in achieving tissue-specific delivery, have further restricted their translational potential. As a result, MDP derivatives have primarily found niche applications as immunomodulatory adjuvants rather than broadly applicable immunotherapeutic agents [98,99,100].

**Figure 3:**
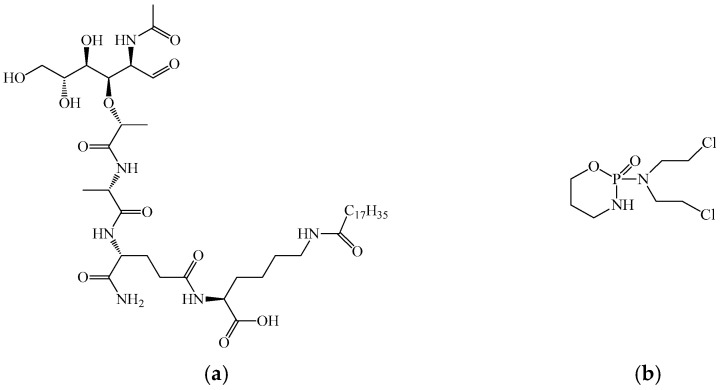
Structure of Romurtide (**a**) and cyclophosphamide (**b**).

At present, only two muramyl dipeptide-based drugs are approved for clinical use: romurtide (Figure 3a) in Japan and Lycopid in Russia. Romurtide (N-acetylmuramyl-L-alanyl-D-isoglutamine-N6-stearoyl-L-lysine, also known as muroctosin) was shown to increase resistance in mice to Staphylococcus aureus, Pseudomonas aeruginosa, Candida albicans, Escherichia coli, opportunistic infection caused by Corynebacterium kutscheri, and to improve survival after intranasal infection with Sendai virus. Romurtide demonstrated a pronounced synergistic protective effect when administered in combination with antibiotics such as cefazolin, gentamicin, and amphotericin B.

Romurtide is also a potent inducer of cytokine synthesis, including IL-1, IL-6, and granulocyte colony-stimulating factor (G-CSF), in monocyte–macrophage cultures. This property formed the basis for investigating its effects on leukopoiesis. On day 7 after subcutaneous administration of 1 mg of romurtide to monkeys, pronounced leukocytosis developed, primarily due to neutrophils. Administration of 100 mg/kg of the drug for eight days following irradiation or cyclophosphamide treatment (Figure 3b) resulted in significantly faster recovery of neutrophil counts in mice compared with controls, accompanied by elevated serum CSF levels. The leukopoietic effect of romurtide is attributed to its capacity to induce CSF synthesis, as well as IL-1, a known potent CSF inducer. Based on these findings, clinical trials were conducted to stimulate leukopoiesis in cancer patients following chemotherapy or radiotherapy. Romurtide effectively restored leukocyte levels, particularly neutrophils, after chemotherapy with cisplatin, vindesine, and mitomycin C, confirming its clinical efficacy in correcting post-chemotherapy leukopenia.

**Figure 4:**
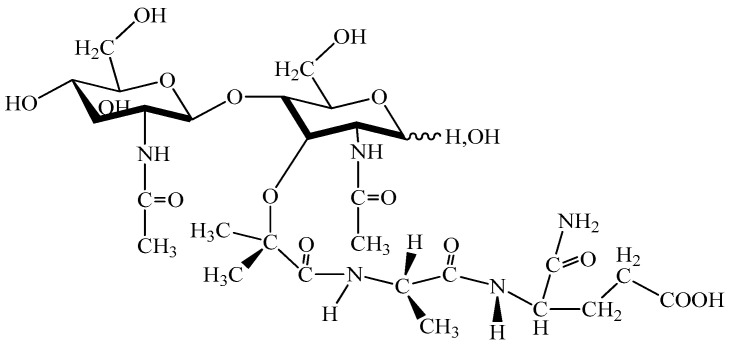
Structure of Lycopid.

Lycopid (Figure 4) is another clinically used leukopoiesis-stimulating agent. During studies of the antitumor drug blastolysin (a hydrolysate of Lactobacillus bulgaricus), researchers isolated an additional bacterial cell wall component, N-acetylglucosaminyl-N-acetylmuramyl-L-alanyl-D-isoglutamine, later named Lycopid. The compound was obtained by direct condensation of a synthetic dipeptide (L-alanyl-D-isoglutamine) with a disaccharide isolated from the cell wall of Micrococcus lysodeikticus.

Lycopid restores leukocyte counts, particularly neutrophils, and activates cells of the monocyte–macrophage lineage. It enhances phagocytosis and microbial killing via activation of lysosomal enzymes and reactive oxygen species production, promotes the elimination of tumor and virus-infected cells, increases HLA-DR expression and antigen presentation, and induces cytokine synthesis, including IL-1, TNF, and colony-stimulating factors [100].

#### 4.3.2. Nucleic Acid-Based Agents

**Figure 5:**
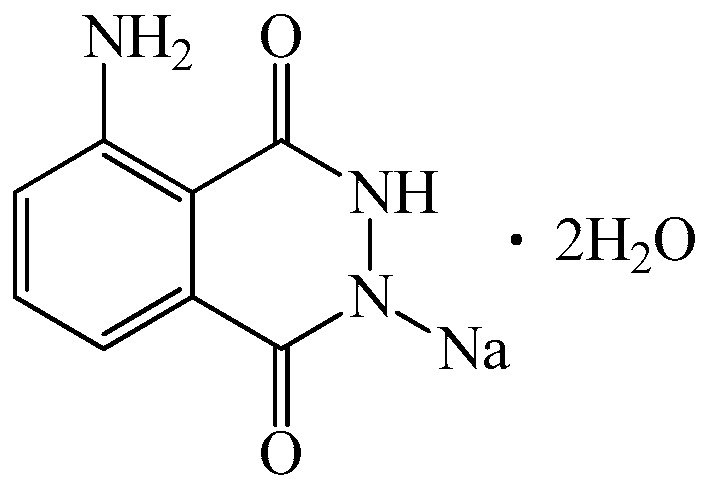
Structure of sodium nucleinate.

Sodium nucleinate (Figure 5) was obtained by hydrolysis and purification from yeast. The compound demonstrated high activity in stimulating bone marrow leukopoiesis and has historically been used as a general immunostimulant. Sodium nucleinate contains a significant amount of nucleic acids and promotes the proliferation of dividing cells, reflecting its ability to enhance hematopoietic activity (immūnomodulators classification).

The drug also stimulates factors of both innate and adaptive immunity, which is consistent with the association of immune response development with active proliferation of T- and B-lymphocytes (classification of immunomodulators). Sodium nucleinate was among the first agents approved for use not only as a leukopoiesis stimulator but also as a nonspecific immune stimulant. Historically, it has been derived from various nucleic acid sources, including native DNA isolated from sturgeon fish. Polydan is a highly purified mixture of sodium salts of DNA and RNA obtained from sturgeon milt, and Ridostin is RNA isolated from baker’s yeast. Many synthetic drugs are also based on nucleic acids, such as poludan—a complex of polyadenylic and uridylic acids. Other compounds in this group include inosine pranobex (isoprinosine), a complex of inosine with acetylamidobenzoic acid, and combinations of methyluracil and riboxin, which consist of hypoxanthine riboside (immunomodulators classification) [101,102,103,104].

However, it is important to consider that both natural and synthetic nucleic acid preparations contain precursors for RNA and DNA and thus may promote cellular proliferation, including that of rapidly dividing cell populations. While this proliferative effect underlies their capacity to stimulate immune cells, it also raises concerns regarding potential oncogenic risks. Exogenous nucleic acids or their metabolic products can, in some contexts, promote DNA replication and cell cycle progression in non-target cells, which theoretically could contribute to the expansion of pre-malignant clones or support growth of transformed cells if regulatory checkpoints are compromised (nucleic acid therapeutics risk considerations). Furthermore, nucleic acid-based therapies and immunostimulatory oligonucleotides are an active area of research in oncology itself, where they are intentionally designed to target cancer cells (e.g., cancer vaccines and gene therapies), but these approaches must carefully balance immune activation with safety to avoid unintended effects on non-malignant tissues (review on nucleic acid immunotherapy and cancer).

Thus, although sodium nucleinate and related compounds can enhance immune responses and leukopoiesis, caution is warranted in their use, because of their capacity to influence proliferative pathways at the genetic level, which could carry theoretical risks of promoting malignant transformation or supporting tumor growth under certain conditions. Preclinical toxicology and long-term surveillance are essential when considering nucleic acid-based immunomodulators to evaluate the balance between immunostimulation and potential oncogenic effects (nucleic acid therapeutic challenges) [105,106,107].

### 4.4. Thymic Peptides and Thymus-Derived Preparations

In the late 1970s, a preparation known as thymosin fraction 5 (TF5) was isolated from bovine thymus tissue [108]. From a physicochemical perspective, TF5 represents a heterogeneous mixture of polypeptides with molecular weights ranging from approximately 1 to 17 kDa [109]. Due to its compositional heterogeneity and the lack of reproducible standardization, TF5 did not achieve widespread clinical application [110,111,112].

The first well-characterized peptide isolated from TF5 was thymosin alpha-1 (Tα1). Its amino acid sequence and physicochemical properties were elucidated, enabling chemical synthesis [113,114]. Tα1 induces lymphocyte differentiation, indirectly stimulates the proliferation and maturation of B lymphocytes, enhances responses to mitogens, increases antibody production, and augments cytokine signaling, including interferons and interleukins such as IL-3 and IL-4. It also enhances cytokine receptor expression and natural killer (NK) cell activity [115,116,117]. Notably, Tα1 is not a thymus-specific hormone, as it is expressed in multiple tissues, and is considered a pleiotropic intracellular regulatory peptide involved in T-cell growth and differentiation, as well as in macrophage and dendritic cell function [118,119,120]. Experimental studies demonstrated that Tα1 promotes endothelial cell migration, angiogenesis, and wound healing, acting as a potent chemoattractant for endothelial cells and monocytes in vitro [121]. In murine models of T-cell lymphoma, administration of Tα1 increased the antitumor activity of dendritic cells, resulting in delayed tumor growth and prolonged survival [122].

Subsequently, interest arose in IRX-2, a naturally derived cytokine mixture with immunostimulatory properties. A combined formulation of Tα1 and IRX-2, termed IRX-3, was developed [123]. IRX-3 was shown to increase CD45RA^+^ leukocyte populations, particularly T lymphocytes, in cancer patients, thereby enhancing the efficacy of immunotherapy. Experimental evidence indicated that Tα1 contributes substantially to the biological activity of IRX-3, significantly increasing proliferative responses of splenic and thymic T cells to mitogens and cytokines [124,125]. Preliminary clinical studies suggested potential applications of Tα1 in autoimmune diseases, oncology (including non-small cell lung cancer and melanoma), viral hepatitis C, vaccine adjuvantation, and HIV infection, with particularly pronounced effects observed in elderly patients. In individuals with AIDS, combination therapies including Tα1 were associated with increases in CD4^+^ T-cell counts [126,127].

Prothymosin alpha. Prothymosin alpha (Pro-Tα) is a 109-amino-acid precursor protein ubiquitously expressed in mammalian cells. It plays an important role in nuclear chromatin remodeling and is localized in both the nucleus and cytoplasm [128,129,130,131]. Proteolytic processing of Pro-Tα by asparaginyl endopeptidase generates Tα1 and Tα11, peptides with immunotropic activity [132]. Synthetic Pro-Tα has been shown in vitro to enhance NK-cell activity, increase antitumor activity of monocytes from melanoma patients, augment T-cell proliferative responses, and restore peripheral lymphocyte counts in cancer patients. Despite its promising biological profile, no approved therapeutic agent based on Pro-Tα has yet been developed [133,134,135].

Other thymic peptides. Other α-thymosins, including thymosin α-5 and α-7, have been identified, although their immunological relevance is limited. Thymosin α-7 demonstrates activity in hematopoietic assays but has not progressed toward clinical development [136,137].

**Figure 6:**
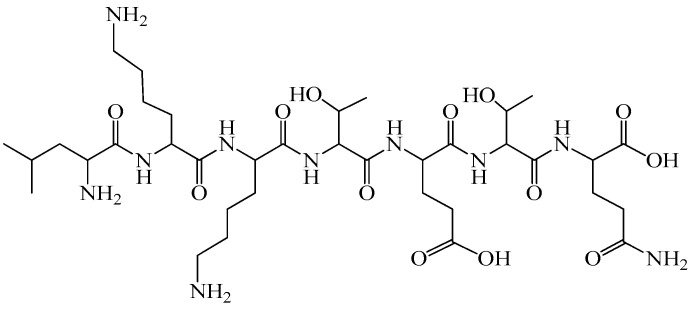
Primary structure of β-thymosin highlighting the conserved actin-binding motif (LKKTET) responsible for G-actin sequestration.

As illustrated in Figure 6, β-thymosins are relatively small (~5 kDa) acidic peptides characterized by an actin-binding motif (LKKTET consensus sequence), which underlies their ability to sequester G-actin and regulate cytoskeletal dynamics. This structural feature explains their intracellular localization and distinguishes them mechanistically from classical secreted thymic hormones. The presence of actin-binding domains accounts for their role in cell motility, angiogenesis, and tissue repair rather than direct lymphoid differentiation [138,139,140,141,142].

**Figure 7:**
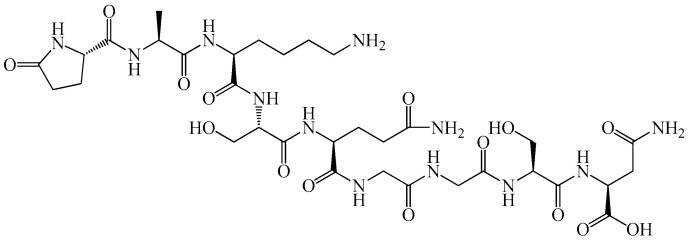
Zinc-dependent nonapeptide structure of thymulin. Zinc coordination is required for biological activity.

Thymulin (Figure 7), a zinc-dependent nonapeptide synthesized by thymic epithelial cells, induces T-cell differentiation and modulates NK-cell and macrophage activity, while also exerting neuroendocrine and neuroprotective effects. Figure 7 highlights the minimal nonapeptide structure of thymulin and its zinc-coordination site, which is essential for biological activity. Zinc binding induces conformational stabilization required for receptor interaction, explaining why thymulin activity is strictly zinc-dependent. This metal-ion-dependent activation distinguishes thymulin from other thymic peptides and links its immunological effects to systemic zinc homeostasis [143,144,145,146,147,148,149].

**Figure 8:**
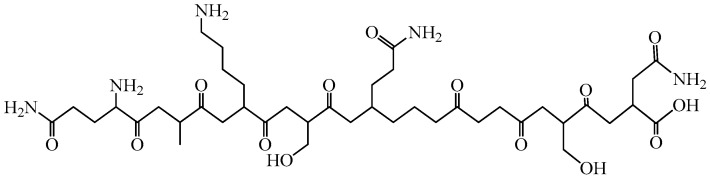
Structure of Thymic humoral factor.

Thymic humoral factor (THF) (Figure 8) and its derivative THF-γ2 demonstrated immunorestorative and antiviral properties in preclinical and limited clinical studies. The structure of thymic humoral factor (THF), shown in Figure 8, reflects its short linear peptide configuration, typical of early thymic extracts. Its limited size and absence of complex tertiary structure suggest receptor-mediated activity through surface immune cell receptors rather than intracellular transcriptional modulation [150,151,152,153,154].

**Figure 9:**
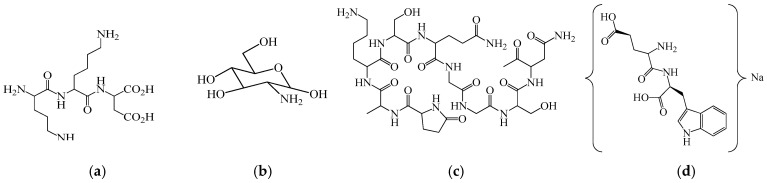
Structural comparison of selected thymic peptides and extracts: (**a**) Thymopentin represents the minimal active fragment of thymopoietin; (**b**) thymomodulin; (**c**) thymalin; (**d**) thymogen.

Figure 9 illustrates the structural diversity of selected thymic peptides and extracts. Notably, thymopentin (TP-5) represents the minimal active pentapeptide fragment of thymopoietin, demonstrating that biological activity can be retained in short functional motifs. In contrast, preparations such as thymomodulin and thymalin are complex peptide mixtures rather than single defined molecular entities, reflecting earlier extraction-based pharmaceutical strategies prior to the era of recombinant protein standardization. Thymopoietin (Figure 9a) and its active pentapeptide fragment thymopentin (TP-5) have been approved in some regions for immunodeficiency and infectious diseases and investigated as adjuncts in oncology [155,156,157,158].

Additional thymic extracts such as thymostimulin, thymic factor X, thymomodulin (Figure 9b), thymalin (Figure 9c), thymogen (Figure 9d), and vilosen showed immunomodulatory effects and regional clinical use, particularly in Eastern Europe, but remain largely outside global clinical guidelines [159,160,161,162,163,164,165,166,167,168,169,170,171].

Collectively, the structural features shown in Figure 6, Figure 7, Figure 8 and Figure 9 highlight a key distinction among thymic peptides: some represent well-defined, short synthetic molecules with characterized sequences (e.g., Tα1, thymopentin), whereas others are heterogeneous extracts with variable composition. This structural heterogeneity has important implications for reproducibility, pharmacokinetics, and regulatory approval, and partly explains the divergent clinical trajectories of these compounds.

Despite extensive preclinical and early clinical research, thymic peptides gradually fell out of mainstream clinical use due to several converging factors. First, many early thymic preparations were heterogeneous extracts derived from animal tissues, leading to batch-to-batch variability, limited standardization, and regulatory challenges. Second, although thymic peptides demonstrated broad immunostimulatory effects, their mechanisms of action were often pleiotropic and insufficiently specific compared with modern targeted immunotherapies, such as monoclonal antibodies and immune checkpoint inhibitors. Third, controlled large-scale randomized clinical trials demonstrating clear efficacy over standard of care were largely lacking, particularly by contemporary evidence-based medicine standards. Finally, advances in molecular immunology shifted therapeutic development toward agents with well-defined receptors, signaling pathways, and biomarkers, reducing interest in thymic peptides as nonspecific immunomodulators. As a result, thymic peptides today are primarily of historical, experimental, or regional clinical interest rather than components of globally adopted immunotherapeutic strategies [172]. Despite historical and regional clinical use of certain thymic preparations, their evidence base remains heterogeneous, with limited large-scale, placebo-controlled randomized trials conducted according to modern regulatory standards. Therefore, thymic peptides should be interpreted primarily as immunomodulatory agents with variable regional clinical integration rather than universally accepted leukopoiesis-stimulating therapies [173].

### 4.5. Plant-Derived Leukopoiesis Modulators

Among plant-derived compounds investigated for potential leukopoiesis-supporting properties, fucoidans represent a structurally diverse group of sulfated polysaccharides currently under experimental evaluation rather than established therapeutic drugs [174]. Fucoidans are long-chain, fucose-rich, sulfated polysaccharides derived primarily from the cell walls of brown seaweeds such as Fucus, Laminaria, and Undaria species. These marine heteropolysaccharides exhibit a broad spectrum of biological activities, including immunomodulatory, anti-inflammatory, anticoagulant, antioxidant, and antitumor effects, and are the subject of intensive preclinical research.

Studies in experimental models, including immunosuppressed mice treated with cytotoxic agents, have shown that certain modified fucoidans and related sulfated oligosaccharides can stimulate hematopoiesis, increasing neutrophils, erythrocytes, and platelets in peripheral blood and bone marrow, often at levels comparable to or exceeding that of recombinant granulocyte colony-stimulating factor (G-CSF) [175]. Additionally, fucoidans can modulate immune cell function by activating macrophages and dendritic cells, promoting cytokine production (including IL-6, TNF-α, and IFN-γ), and enhancing natural killer cell proliferation and cytotoxicity, which may indirectly support leukopoiesis and immune recovery [176,177,178,179,180].

However, the biological effects of fucoidans are highly dependent on molecular weight, degree of sulfation, and structural features, with significant variation in activity between sources and extraction methods; this structural diversity complicates standardization and drug development. Although fucoidans have shown promising immunostimulatory and supportive hematopoietic activity in vitro and in animal models, rigorous clinical evidence in humans remains limited, and as of now no fucoidan-based compound has received broad regulatory approval as a leukopoiesis stimulant.

Importantly, despite promising immunomodulatory and hematopoietic-supportive effects observed in preclinical models, fucoidans should not currently be regarded as an established pharmacological class of leukopoiesis-stimulating agents. Their substantial structural heterogeneity, variability in extraction and purification methods, and limited high-quality clinical evidence restrict their therapeutic classification. At present, fucoidans are more appropriately considered experimental biologically active compounds with potential immunoregulatory properties rather than validated therapeutic agents. Standardization challenges, batch-to-batch variability, and the absence of large randomized controlled trials further limit their integration into evidence-based clinical practice. Rigorous pharmacokinetic characterization, dose optimization, and regulatory-grade manufacturing processes would be required before fucoidans could be positioned as therapeutic drugs [181,182].

### 4.6. Chemically Synthesized Small Molecules and Emerging Targets

**Figure 10:**
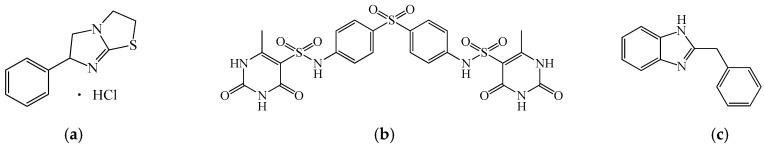
Structure of Levamisole (**a**), Diucifon (**b**) and Dibazol (**c**).

Levamisole (2,3,5,6-tetrahydro-6-phenylimidazo [2,1-b]thiazole hydrochloride; Figure 10a) represents one of the earliest examples of drug repurposing in immunotherapy. Originally introduced as an anthelmintic agent for the treatment of ascariasis, levamisole was later shown to exert immunomodulatory effects, primarily targeting immature and functionally suppressed T lymphocytes. At the molecular level, levamisole modulates intracellular signaling through stimulation of cyclic AMP (cAMP) formation, thereby increasing T-cell responsiveness to thymic factors. Functionally, this results in enhanced protein synthesis, increased blast transformation of lymphocytes, and potentiation of T-helper cell activity. In parallel, levamisole activates macrophage functions, including phagocytosis and chemotaxis, leading to increased antibody production. Notably, levamisole exhibits target specificity toward innate immune effector pathways, as it activates the alternative complement cascade with rapid generation of C5a both in vitro and in vivo, followed by granulocyte aggregation and recruitment, highlighting its role as an early modulator of innate–adaptive immune crosstalk [183].

Diucifon (N,N’-(sulfonylbis(4,1-phenylene))bis(6-methyl-2,4-dioxo-1,2,3,4-tetrahydropyrimidine-5-sulfonamide, Figure 10b), a derivative of diaminodiphenyl sulfone conjugated with two methyluracil residues, was originally developed to improve the tolerability of anti-leprosy therapy. Subsequent studies revealed its repurposing potential as a T-cell-directed immunostimulant. Diucifon selectively enhances the activity of IL-2-producing cells, resulting in amplified IL-2 secretion and downstream activation of cytotoxic T lymphocytes and natural killer cells. This cytokine-centric mechanism confers relative specificity for the T-cell arm of adaptive immunity, positioning diucifon as a prototype for small-molecule modulators of cytokine-driven immune activation [184].

Dibazol (2-benzyl-1*H*-benzo[d]imidazole, Figure 10c) exemplifies an immunomodulator acting through second-messenger signaling pathways. Its immunostimulatory activity is mediated by regulation of the intracellular cGMP/cAMP balance, favoring increased cGMP levels in immune cells. This shift promotes the proliferation of mature, antigen-experienced T and B lymphocytes, enhances cooperative cellular interactions, and activates effector immune responses. Dibazol also increases post-vaccination antibody production, enhances phagocytic and bactericidal activity of leukocytes, and exhibits interferonogenic properties. Importantly, its effects are predominantly preventive rather than therapeutic in acute infections, underscoring the importance of temporal targeting of immune signaling pathways.

**Figure 11:**
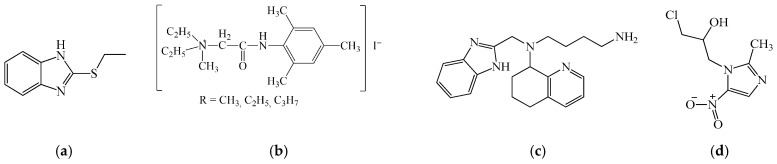
Structure of Bemitil (**a**), the ionic Trimecaine derivative (**b**), Mavorixafor (**c**) and Ornidazole (**d**).

Bemitil (2-(ethylthio)-1*H*-benzo[d]imidazole, Figure 11a) represents a multifunctional example of metabolic-immune repurposing. In addition to leukopoiesis-stimulating activity, bemitil exhibits hepatoprotective, antioxidant, and adaptogenic properties. Immunologically, it restores the CD4^+^/CD8^+^ T-cell ratio, reduces pathological hypergammaglobulinemia, and decreases circulating immune complexes. Notably, bemitil demonstrates selective cytokine reprogramming, suppressing excessive IL-1 and IFN-γ production while enhancing IFN-α and regulatory cytokines, thereby reducing the risk of immunopathological reactions in chronic inflammatory conditions such as viral hepatitis [185].

An ionic trimecaine derivative (TIC, N, N-diethyl-N-(2-(mesitylamino)-2-oxoethyl) propan-1-aminium iodide, Figure 11b) highlights the concept of lineage-specific hematopoietic targeting. In models of cyclophosphamide-induced pancytopenia, TIC selectively stimulated recovery of transitional, follicular, marginal zone, and germinal center B cells without affecting early B-cell progenitors (Pro-B and Pre-B stages). This restricted activity profile represents a favorable therapeutic characteristic, minimizing the risk of uncontrolled progenitor expansion while promoting functional immune reconstitution [186,187].

Mavorixafor (N1-((1*H*-benzo[d]imidazol-2-yl)methyl)-N1-(5,6,7,8-tetrahydroquinolin-8-yl)butane-1,4-diamine, Figure 11c) is a paradigmatic example of target-specific immunotherapy, acting as a selective antagonist of the chemokine receptor CXCR4. By blocking CXCL12 (stromal cell-derived factor-1) binding, mavorixafor disrupts pathological bone marrow retention of leukocytes, leading to mobilization of mature neutrophils and lymphocytes into the peripheral blood. This mechanism underlies its clinical application in patients with WHIM syndrome and exemplifies successful translation of chemokine-axis targeting into clinical immunology [188].

Several antimicrobial agents have also gained attention as emerging immunometabolic modulators. Ornidazole (1-chloro-3-(2-methyl-5-nitro-1*H*-imidazol-1-yl)propan-2-ol, Figure 11d) modulates macrophage activation and inflammatory signaling, suppressing IL-6 and TNF-α while upregulating anti-inflammatory mediators such as IL-1Ra [189].

**Figure 12:**
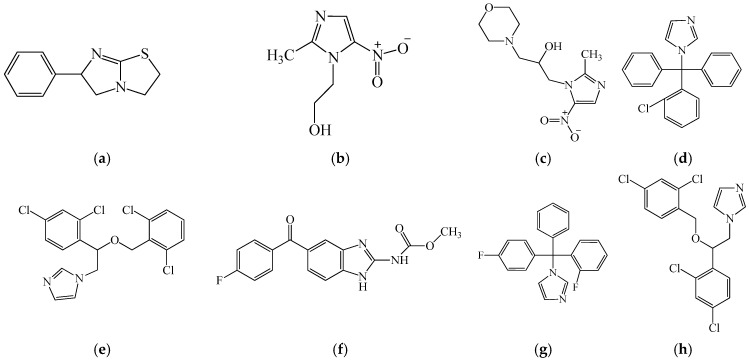
Structure of small molecules: (**a**) Tetramisole; (**b**) the ionic Trimecaine derivative; (**c**) Mavorixafor; (**d**) Ornidazole; (**e**) Isoconazole; (**f**) Flubendazole; (**g**) Flutrimazole; (**h**) Miconazole.

Tetramisole (6-phenyl-2,3,5,6-tetrahydroimidazo [2,1-b]thiazole, Figure 12a). Triclabendazole and the combination of tetramizole and oxyclosanide demonstrate a strong efficacy against fasciolosis in sheep [190].

Metronidazole (2-(2-methyl-5-nitro-1*H*-imidazol-1-yl)ethanol, Figure 12b) and morinidazole (1-(2-methyl-5-nitro-1*H*-imidazol-1-yl)-3-morpholinopropan-2-ol, Figure 12c) similarly demonstrate immunomodulatory effects beyond antimicrobial activity, influencing leukocyte function, epithelial barrier integrity, and cytokine production [191,192].

Azole antifungals represent a particularly active area of oncology-oriented drug repurposing. Clotrimazole (1-((2-chlorophenyl)diphenylmethyl)-1*H*-imidazole, Figure 12d) targets glycolytic enzymes, notably hexokinase-2, thereby linking metabolic stress to mitochondrial apoptosis and enhanced antigen presentation by dendritic cells, which results in improved T-cell activation [193].

Isoconazole (1-(2-((2,6-dichlorobenzyl)oxy)-2-(2,4-dichlorophenyl)ethyl)-1*H*-imidazole; structure shown in Figure 12e) has been reported to induce ROS-mediated cytotoxicity in hepatocellular carcinoma cells, while concomitantly activating the NRF2–metallothionein axis as part of an adaptive cellular response [194]. Flubendazole (methyl (5-(4-fluorobenzoyl)-1*H*-benzo[d]imidazol-2-yl)carbamate; Figure 12f) further exemplifies drug repurposing in immune-oncology. It downregulates PD-1 expression, enhances CD3^+^ T-cell infiltration, and modulates transcriptional networks associated with T-cell differentiation and effector function [195]. Flutrimazole (1-((2-fluorophenyl)(4-fluorophenyl)(phenyl)methyl)-1*H*-imidazole; Figure 12g) and Miconazole (1-(2-((2,4-dichlorobenzyl)oxy)-2-(2,4-dichlorophenyl)ethyl)-1*H*-imidazole; Figure 12h) likewise demonstrate pronounced antifungal efficacy in rabbit and human skin infected with fungal pathogens [196,197]. These compounds are proposed to support hematopoietic recovery via cytotoxic, anti-inflammatory, and microenvironment-stabilizing mechanisms.

**Figure 13:**
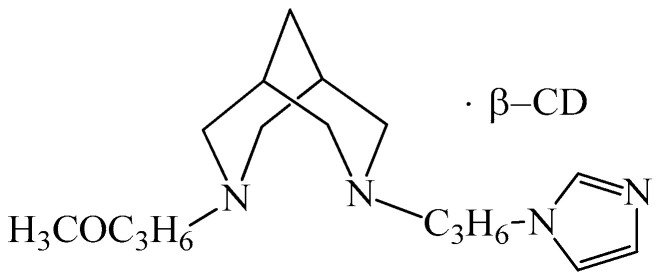
Structure of 3-(3-(1*H*-imidazol-1-yl)propyl)-7-(3-methoxypropyl)-3,7-diazabicyclo [3.3.1]nonane.

It was found that the bicyclic imidazole derivative (Figure 13) has the ability to activate polyamine oxidase in liver lysates and is toxic to tumor cells. In addition, it demonstrates the promotion of WI-38 and HepG2 viability after cell incubation with PAs [198].

Collectively, these compounds illustrate how repurposing of structurally diverse small molecules—originally developed as antiparasitic, antimicrobial, or metabolic agents—can uncover unexpected target specificity and novel molecular mechanisms relevant to leukopoiesis, immune reconstitution, and cancer immunotherapy.

## 5. Clinical Positioning and Safety Considerations

Leukopoiesis-stimulating strategies are most commonly applied in clinical contexts characterized by impaired bone marrow function or excessive leukocyte depletion. The etiologies of such conditions are heterogeneous and include cytotoxic chemotherapy and radiotherapy, hematologic malignancies (e.g., acute leukemias, myelodysplastic syndromes), bone marrow infiltration by solid tumors, autoimmune-mediated marrow suppression, drug-induced agranulocytosis, severe infections, congenital neutropenia syndromes, and aplastic anemia. In addition, sepsis and chronic inflammatory states may induce secondary bone marrow exhaustion or dysregulated myelopoiesis. These diverse etiologies differ substantially in their pathophysiological mechanisms—ranging from direct stem cell toxicity and microenvironmental disruption to immune-mediated destruction—thereby influencing the choice and effectiveness of leukopoiesis-stimulating interventions. Importantly, the risk–benefit balance of leukopoiesis-stimulating therapies varies depending on etiology. For example, in chemotherapy-induced neutropenia, short-term stimulation may reduce infection risk, whereas in clonal hematopoietic disorders, excessive stimulation could theoretically exacerbate malignant proliferation. Common etiologies of leukopenia include impaired production due to bone marrow disorders, autoimmune-mediated destruction, drug-induced suppression, nutritional deficiencies, infection, and hypersplenism—each demanding tailored diagnostic and therapeutic strategies [199].

Across all classes, currently approved therapeutic agents show that immune manipulation is clinically feasible. Table 3 demonstrates comparison of major classes of therapeutic agents with respect to mechanisms of action, clinical implementation, safety profiles, and unmet therapeutic needs. While several agents are clinically established, most classes exhibit limitations related to specificity, safety, or delivery, underscoring the demand for next-generation precision immunotherapies.

## 6. Limitations of the Review

This review has several limitations. First, the immunomodulatory agents discussed represent highly heterogeneous classes with distinct molecular targets, mechanisms of action, and clinical contexts, which limits direct comparability across studies. Second, there is a substantial lack of head-to-head clinical trials directly comparing different immunomodulatory strategies, making it difficult to draw definitive conclusions regarding relative efficacy or safety. Finally, significant translational gaps remain between preclinical findings and clinical application, as many promising agents are supported primarily by in vitro or animal data, with limited validation in large, well-controlled human studies.

## 7. Future Directions in Leukopoiesis-Targeted Therapy

Future advances in leukopoiesis-targeted therapy are expected to move beyond uniform stimulation of myeloid cell production toward more precise, context-dependent immune modulation. One promising direction is personalized leukopoiesis stimulation, in which therapeutic interventions are tailored to individual hematopoietic and inflammatory profiles. Advances in single-cell transcriptomics, immune phenotyping, and biomarker-guided stratification may enable selective activation of specific leukocyte lineages while avoiding excessive or dysregulated immune responses.

Another key area is the development of combination strategies that integrate leukopoiesis-stimulating agents with immunotherapies, antimicrobial treatments, or anti-inflammatory drugs. Such approaches aim to enhance immune recovery while simultaneously shaping functional immune responses, particularly in oncology, post-chemotherapy settings, and chronic inflammatory conditions. Rational combinations may also allow dose reduction in individual agents, thereby improving safety profiles.

Targeting the hematopoietic niche represents an emerging paradigm in leukopoiesis modulation. Rather than directly stimulating progenitor cells, niche-focused therapies seek to modulate bone marrow stromal cells, cytokine gradients, and chemokine axes that regulate hematopoietic stem and progenitor cell behavior. By influencing the microenvironment that governs leukocyte production, niche-targeting strategies may provide more durable and physiologically balanced immune reconstitution.

Gene-based approaches represent an emerging frontier in leukopoiesis-modulating strategies. Advances in gene editing technologies, including CRISPR/Cas9-mediated correction of inherited bone marrow failure syndromes and ex vivo modification of hematopoietic stem cells (HSCs), offer the potential for durable restoration of leukocyte production. Gene therapy strategies are currently being explored for congenital neutropenia, severe combined immunodeficiency, and other monogenic disorders affecting hematopoiesis. In addition, vector-mediated modulation of transcription factors or cytokine signaling pathways may enable long-term correction of lineage imbalances without continuous pharmacologic stimulation. However, challenges including off-target effects, insertional mutagenesis, long-term safety, manufacturing complexity, and high cost currently limit widespread clinical implementation. Emerging gene therapy technologies, including CRISPR/Cas9 and base editors, show promise in preclinical and early clinical settings for correction of inherited hematopoietic defects and leukopenia syndromes, potentially enabling durable restoration of leukocyte production [208,209].

An additional emerging direction involves metabolic and microenvironment-oriented support of hematopoiesis. Increasing evidence indicates that hematopoietic stem cell (HSC) function is tightly coupled to nutrient availability, redox balance, and lipid metabolism within the bone marrow niche. Supplementation with specific metabolites such as serine, phosphate, and cholesterol has been shown in experimental models to enhance HSC survival and regenerative capacity following myelosuppressive injury by supporting nucleotide biosynthesis, membrane organization, and intracellular signaling stability. Serine contributes to one-carbon metabolism and nucleotide synthesis, which are essential for proliferating progenitor cells, whereas cholesterol and lipid homeostasis regulate membrane organization and growth factor receptor signaling within the niche microenvironment [210]. Similarly, niche-associated cytokines such as insulin-like growth factor-1 (IGF-1) and plant-derived bioactive compounds including ferulic acid have demonstrated the ability to promote hematopoietic recovery through modulation of oxidative stress, inflammatory signaling, and stem cell maintenance pathways. Nutritional and metabolic modulation, including supplementation with antioxidants such as ferulic acid, enhances HSC maintenance and reduces susceptibility to ferroptosis following myelosuppressive injury, thereby supporting hematopoiesis at homeostasis and in damage settings [211]. Moreover, local niche-associated factors such as insulin-like growth factor-1 (IGF-1) have been shown to coordinate activation, proliferation, and regeneration of hematopoietic stem cells after injury, with IGF-1 signaling promoting functional expansion and protection of HSCs via downstream pathways in preclinical models [212]. These approaches represent a conceptual shift from direct lineage-restricted stimulation toward restoration of physiological niche homeostasis. However, most evidence remains preclinical, and controlled clinical validation will be required before integration into routine practice.

Finally, there is a growing demand for safer alternatives to colony-stimulating factors (CSFs). While CSFs remain effective, their broad and sometimes pro-inflammatory effects limit long-term or repeated use. Next-generation approaches—including selective pathway modulators, engineered peptides, nucleic acid-based regulators, and targeted delivery systems—aim to decouple leukocyte expansion from systemic inflammation. These innovations may ultimately enable controlled, lineage-specific leukopoiesis with improved tolerability and reduced risk of adverse immune activation.

The future landscape of leukopoiesis-stimulating therapies is likely to evolve toward precision-based modulation of hematopoiesis. Rather than uniform stimulation with colony-stimulating factors, emerging strategies aim to integrate molecular profiling, niche-targeted interventions, transcriptional regulation, cellular therapies, and potentially gene editing approaches. Personalized assessment of marrow reserve, inflammatory status, clonal hematopoiesis, and immune context may guide selection between short-term pharmacologic stimulation, stromal support strategies, or durable genetic correction. Such stratified approaches may reduce adverse effects associated with excessive or unbalanced leukocyte expansion while improving long-term hematopoietic stability.

## 8. Conclusions

Leukopoiesis-stimulating therapies represent a critical component of modern biomedical practice, particularly in the management of leukopenia associated with oncological treatments, infectious diseases, immune dysregulation, and iatrogenic conditions. While colony-stimulating factors remain the cornerstone of clinical leukopoiesis stimulation, their limitations have highlighted the need for alternative and complementary strategies.

Advances in the understanding of leukopoiesis regulation have revealed that leukocyte production is controlled by a complex, multi-level network involving bone marrow niche interactions, cytokine signaling, transcriptional regulation, chemokine gradients, and immune-mediated feedback mechanisms. This complexity provides multiple therapeutic entry points, enabling pharmacological and biotechnological targeting of leukopoiesis beyond conventional growth factor administration.

As summarized in Table 1 and Table 3, current leukopoiesis-modulating approaches differ substantially in their molecular targets, level of hematopoietic regulation, and degree of clinical validation. In addition to established cytokine-based therapies, a growing number of endogenous peptides, immune modulators, small molecules, and niche-targeting agents demonstrate the potential to stimulate leukopoiesis through more selective and physiologically relevant mechanisms. However, many of these approaches remain at the preclinical or early translational stage, underscoring the need for rigorous clinical evaluation.

Future progress in the field of leukopoiesis-targeted therapy will likely depend on the development of strategies that integrate mechanistic specificity with clinical safety and efficacy. Personalized approaches, combination therapies, and interventions targeting early regulatory nodes of hematopoiesis may offer more durable and controlled restoration of leukocyte production. Continued translational research bridging molecular insights with clinical application is essential for advancing safer and more effective leukopoiesis-stimulating therapies for patients with diverse forms of leukopenia.

## Figures and Tables

**Table 1:** Major Pharmacological and Biotechnological Strategies Targeting Leukopoiesis.

Therapeutic Class	Representative Agents	Primary Molecular Targets/Mechanisms	Level of Leukopoiesis Regulation	Evidence Level	Clinical Status	Key Limitations
Bone marrow-derived regulatory peptides	MP-1, MP-2, MP-3, MP-4, MP-5, MP-6	Endogenous peptide-mediated modulation of immune cell proliferation and differentiation	Progenitor and immune differentiation level	Preclinical	Experimental/veterinary use	Limited clinical translation, lack of standardized formulations
Granulocyte colony-stimulating factors (G-CSF)	Filgrastim, Pegfilgrastim, Lenograstim	Activation of G-CSF receptor, stimulation of granulocytic proliferation and maturation	Late-stage differentiation (granulopoiesis)	Clinical (high)	Widely approved	Bone pain, splenomegaly, lineage restriction, limited efficacy in refractory neutropenia
Granulocyte–macrophage CSF (GM-CSF)	Sargramostim	Activation of GM-CSF receptor, expansion of granulocyte–macrophage progenitors	Progenitor and differentiation level	Clinical (moderate)	Approved (selected indications)	Pro-inflammatory effects, variable clinical response
Thymic peptides	Thymosin α1, Thymalin, Thymogen	Modulation of T-cell maturation, cytokine signaling and immune homeostasis	Immune maturation and regulation	Clinical (low–moderate)	Approved (regional)	Indirect leukopoiesis stimulation, heterogeneous clinical efficacy
Muramyl dipeptide derivatives	Romurtide, Lycopid	Activation of innate immune signaling, induction of cytokines including CSFs	Immune-mediated regulation of leukopoiesis	Clinical (regional)/preclinical	Approved (regional)/experimental	Pyrogenicity, inflammatory adverse effects
Nucleic acid-based agents	Sodium nucleinate, Poludan, Inosine pranobex	Provision of nucleic acid precursors, stimulation of immune cell proliferation	Broad proliferative regulation	Clinical (low)	Approved (regional)	Non-selective cell proliferation, potential oncological risks
Plant-derived immunomodulators	Fucoidans	Modulation of cytokine production and immune activation	Immune regulatory level	Preclinical	Experimental	Variability of composition, limited clinical evidence
CXCR4 antagonists	Mavorixafor	Disruption of CXCL12–CXCR4 axis, mobilization of leukocytes from bone marrow niche	Stem and progenitor cell mobilization	Clinical (moderate)	Approved (rare diseases)	Narrow indication spectrum, long-term safety unknown
Chemically synthesized small molecules (repurposed)	Levamisole, Bemitil	Modulation of immune signaling pathways and cellular metabolism	Immune activation and differentiation	Clinical (low–moderate)	Approved/repurposed	Limited selectivity, off-target effects
Emerging small-molecule and nanotechnology-based agents	Imidazole derivatives, peptide-loaded nanoparticles	Targeting transcription factors, niche interactions, and differentiation signaling	Multi-level regulation	Preclinical	Preclinical/early experimental	Insufficient clinical validation

**Table 2:** Structure of primary myelopeptides.

No.	Myelopeptides	Composition	Functions
1	MP-1	Phe-Leu-Gly-Phe-Pro-Thr	Restoration of antibody genesis
2	MP-2	Leu-Val-Val-Tyr-Pro-Trp	Antitumor immunity
3	MP-3	Leu-Val-Cys-Tyr-Pro-Gln	Effect on the monocytic unit
4	MP-4	Phe-Arg-Pro-Arg-Ile-Met-Thr-Pro	Cell differentiation factor, hematopoiesis regulator
5	MP-5	Val-Val-Tyr-Pro-Asp	Cell differentiation factor
6	MP-6	Val-Asp-Pro-Pro	Cell differentiation factor

**Table 3:** Comparative overview of major classes of therapeutic agents.

Therapeutic Class	Mechanism of Immune Modulation	Established Clinical Use	Experimental/Translational Status	Safety/Adverse Effects	Rationale for Development of New Agents	Ref.
Bone marrow-derived regulatory peptides	Peptide-mediated regulation of innate and adaptive immune responses	Limited regional or experimental clinical use	Development as immunomodulatory and antimicrobial peptides; vaccine adjuvants	Generally low toxicity; limited human safety data	Insufficient potency and lack of large randomized clinical trials	[200]
GM-CSF (Granulocyte–macrophage CSF)	Stimulation of myeloid differentiation, antigen presentation, macrophage and dendritic cell activation	Approved for myeloid recovery after chemotherapy and hematopoietic stem cell transplantation	Investigated as vaccine adjuvant and in cancer immunotherapy combinations	Pro-inflammatory effects, fever, capillary leak syndrome; context-dependent pro-tumorigenic activity	Broad and context-sensitive immune effects limit predictability and safety	[201]
Muramyl dipeptide (MDP) derivatives	Activation of innate immunity via NOD2 signaling and macrophage stimulation	Approved derivative (mifamurtide) for osteosarcoma adjuvant therapy in selected regions	Development of novel analogs with improved selectivity	Fever, chills, cytokine-mediated inflammation; narrow therapeutic window	Excessive innate immune activation and systemic inflammatory toxicity	[202]
Thymic peptides	Regulation of T-cell maturation, differentiation, and immune homeostasis	Used regionally for immune rehabilitation and secondary immunodeficiency	Investigated in aging immunity, viral infections, and oncology supportive care	Favorable safety profile; mild systemic or injection-site reactions	Non-specific immune effects and limited molecular targeting	
Nucleic acid-based agents (siRNA, antisense, mRNA)	Gene-level modulation of immune signaling pathways and immune cell programming	Multiple oligonucleotide drugs approved; mRNA platforms clinically validated	Autoimmune disease modulation, cancer immunotherapy, immune reprogramming	Innate immune activation, delivery-related toxicity, off-target gene effects	Need for improved delivery, tissue specificity, and immune control	[203]
Plant-derived immunomodulators	Modulation of cytokine signaling (e.g., NF-κB, MAPK pathways) via phytochemicals	Used mainly as dietary supplements or adjunct therapies	Preclinical and early clinical studies in inflammatory and autoimmune models	Low toxicity; variability in bioavailability and drug–drug interactions	Limited potency and reproducibility; need for standardized derivatives	[204]
CXCR4 antagonists	Disruption of CXCR4–CXCL12 axis; modulation of immune cell trafficking	Approved for hematopoietic stem cell mobilization (plerixafor)	Oncology and metastasis-targeting strategies under investigation	Generally well tolerated; concerns regarding chemokine network perturbation	Limited indications and need for refined targeting in cancer and chronic inflammation	[205]
Chemically synthesized small molecules (repurposed)	Targeting immune signaling pathways (e.g., TLRs, STING, checkpoint-related pathways)	Several agents in clinical trials; some repurposed from non-immune indications	Broad exploration in oncology and immune-mediated diseases	Off-target effects and systemic toxicity	Requirement for higher specificity and improved therapeutic index	[206]
Emerging small-molecule and nanotechnology-based agents	Targeted delivery, controlled release, and immune cell-specific modulation	Early clinical evaluation for selected nanoformulations	RNA delivery systems, tumor-targeted immunomodulation, combination platforms	Nanotoxicity, biodistribution, and long-term accumulation concerns	Designed to overcome delivery, specificity, and safety limitations of existing agents	[207]

## Data Availability

The datasets used and/or analyzed during the present study are available from the corresponding author on reasonable request.

## Supplementary Materials

The following supporting information can be downloaded at: https://www.mdpi.com/article/10.3390/biomedicines14030624/s1, Table S1: Drugs approved for therapy, characteristics, and mechanisms of action, advantages, limitations and application. References [213,214,215,216,217,218,219,220,221,222,223,224,225,226,227,228,229,230,231,232,233,234,235,236,237,238,239,240,241,242,243,244,245,246,247,248,249,250,251,252,253,254] are cited in supplementary materials.

## Abbreviations

The following abbreviations are used in this manuscript:

BM-MSC	Bone marrow multipotent mesenchymal stromal cells
CSF	Colony-stimulating growth factor
DNA	Deoxyribonucleic acid
GM-CSF	Granulocyte-macrophage colony-stimulating growth factor
HGF	Hepatocyte growth factor
HLA	Human leukocyte antigens
IDO	Indole-2.3-deoxygase
IF	Interferons
IGF-I	Insulin-like growth factor
IL	Interleukin
MDP	Muramyl dipeptide
MP	Myelopeptide
NK	Natural killer
PGE-2	Prostaglandin E2
Pro-T-alpha	Prothymosin alpha
PU.1	Purine-rich box protein 1
GATA-1	Erythroid transcription factor
RNA	Ribonucleic acid
SCF	Stem cell factor
T-alpha-1	Thymosin alpha-1
TF5	Thymosin fraction 5
TFX	Thymic factor X
THF	Thymic humoral factor
TNF	Tumor necrosis factor
