# Supplementary material for: Targeting Leukopoiesis: Pharmacological and Biotechnological Strategies for the Treatment of Leukopenia
Source: Biomedicines. 2026 Mar 11;14(3):624. doi: 10.3390/biomedicines14030624 (PMC13023911; doi:10.3390/biomedicines14030624)
Supplement: Supplementary file 1 [file biomedicines-14-00624-s001.zip › Supplementary - Table.pdf]

Table S1. Drugs approved for therapy, characteristics, and mechanisms of action, advantages, limitations and applications

	Generic and trade names of drugs	Material / Model/ {DrugBank ID}	Empirical formula / MW	Targeting mechanism	Advantages	Limitations / side effects	Application	Clinical status	Ref.
1	BM-MSCs (Ryoncil®, Remestemcel-L®)	The drugs of bone marrow origin	NA	Inhibition of T-cell activation (T-cells mediate the inflammation, cell damage and organ damage associated with acute GVHD)	- Improving survival outcomes with SR-αGVHD	- Hypersensitivity and acute infusion reactions;<br>- Transmission of infectious agents;<br>- Ectopic tissue formation	- Treatment of steroid-refractory acute graft-versus-host disease	Tested in humans	[213]
2	Filgrastim (Accofil®, Filgrastim Hexal®, Granix®, Grastofil®, Neupogen®, Nivestim®, Nivestym®, Nypozi®, Ratiograstim®, Releuko®, Tevagrastim®, Zarxio®, Zarzio®)	The drugs of bone marrow origin, colony-stimulating growth factor, Leukocyte growth factor<br><br>{DB00099}	$C_{845}H_{1339}N_{223}O_{243}S_9$<br><br>18 800.0 Da	Granulocyte colony-stimulating factor receptor, which stimulates the proliferation, maturation of neutrophil progenitors and functional end-cell activation	- The release of neutrophils from the bone marrow into the blood to reduce the incidence of infection;<br>- manage neutropenia	- Possible ARDS, - myelodysplastic syndrome, kidney damage (Glomerulonephritis)	- Induce the production of granulocytes<br>- Lower infection risk after myelosuppressive therapy	Phase IV clinical trials	[214]
3	Lenograstim (Granocyte®)	The drugs of bone marrow origin, colony-stimulating factor<br><br>{DB13144}		Granulocyte colony-stimulating factor receptor, it assists neutrophil recovery in patients undergoing bone marrow transplantation and stimulates the production of peripheral blood stem cells	- Reduction of the haematological toxicity of myelosuppressive chemotherapy with malignant disease;<br>- Enhancing of neutrophil recovery	- Possible general aches and pains	- Neutropenia caused by chemotherapy;<br>- Bone marrow transplantation;<br>- Peripheral progenitor cell transplantation;<br>- Severe congenital neutropenia	Phase IV clinical trials	[215]
4	Pegfilgrastim (Cegfila, Fulphila, Fylnetra, Grasustek, Neulasta, Nyvepria, Pelgraz, Pelmeg, Stimufend, Udenyca, Ziextenzo®)	The drugs of bone marrow origin, colony-stimulating growth factor, Leukocyte growth factor<br><br>{DB00019}	$C_{845}H_{1343}N_{223}O_{243}S_9$<br><br>39 000.0 Da	Human granulocyte colony-stimulating growth factor, which stimulates the growth of white blood cells, neutrophils	- Prevention febrile neutropenia by increasing the body's neutrophil level;<br>- inducing neutrophil proliferation, differentiation and maturation	- Possible bone pains, splenic rupture, acute respiratory distress syndrome, sickle cell disorders, glomerulonephritis, thrombocytopenia, capillary leak syndrome, myelodysplastic syndrome	- Neutropenia caused by radiation or chemotherapy	Phase IV clinical trials	[216]
5	Eflapegrastim (Rolvedon®)	The drugs of bone marrow origin, colony-stimulating growth factor, Leukocyte growth factor<br><br>{DB15001}	NA<br><br>72 000.0 Da	Human granulocyte colony-stimulating growth factor, which binds to G-CSF receptors on myeloid progenitor cells and neutrophils to result in neutrophil differentiation, proliferation, migration and survival	- Increasing absolute neutrophil count;<br>- Higher serum and bone marrow concentrations significantly shorter than duration of neutropenia	- Possible capillary leak syndrome, shortness of breath and kidney problems	- Neutropenia and lower infection risk after myelosuppressive therapy and cancer	Phase IV clinical trials	[217]

6	Efbemalenograstim alfa (Ryzneuta®)	The drugs of bone marrow origin, colony-stimulating growth factor, Leukocyte growth factor<br><br>{DB18704}	NA	It stimulates proliferation, differentiation, commitment and end-cell functional activation by binding to specific receptors	- Effectiveness against CIN;<br>- Less neutropenia in later cycles;<br>- Safety of treatment with low incidences of serious AEs and TEAEs<br>- Extension circulation half-life by increasing the hydrodynamic diameter of the fusion protein	- Possible nausea, angioedema, urticaria, hypertension and bone pain	- The prevention of infection in cancer patients or Neutropenia caused by chemotherapy;	Phase III clinical trials	[218]
7	Ancestim (Stemgen®)	The drugs of bone marrow origin, Haematopoietic growth factor<br><br>{DB09103}	$C_{1662}H_{2650}N_{422}O_{512}S_{18}$<br><br>18 540.0 Da	Mast/Stem cell growth factor, which is alone an unable to increase peripheral blood progenitor cell so it has to be administered with filgrastim. It binds to the c-KIT and activates of signaling the RAS/EPK, P13-kinase and JAK/STAT	- Increasing of availability of stem cells for transplantation;<br>- Expansion of circulating peripheral blood progenitor cells, erythroid burst-forming units to stimulate its proliferation, commitment and functional activation	- Possible capillary leak syndrome, hypotension, hypoalbuminemia and edema	- Increasing of the number and mobilization of peripheral blood progenitor cells	Phase II clinical trials	[219]
8	Sargramostim (Leukine®)	The drugs of bone marrow origin, Leukocyte growth factor, Haematopoietic growth factor<br><br>{DB00020}	$C_{639}H_{1006}N_{168}O_{196}S_{8}$<br><br>14 434.5 Da	Granulocyte-macrophage colony-stimulating factor receptor (GM-CSF-R-alpha or CSF2R), which stimulates a JAK 2 STAT1/STAT3 signal transduction pathway. It leads to production of hemopoietic cells and neutrophils.	- Improvement anti-tumor responses in melanoma;<br>- Reduction the risk for GI irAEs and pulmonary adverse events;<br>- Attenuation anticancer effect of other agents	- Possible capillary leak syndrome and pleural or pericardial effusion	- Increasing of immune cell production to prevent infections after radiation or chemotherapy	Phase IV clinical trials	[220]
9	Romurtide (Nopia®, ImmunoBoost®, NeutroStim®)	The drugs of microbial origin, synthetic muramyl dipeptide<br><br>{NA}	$C_{43}H_{78}N_{6}O_{13}$<br><br>887.11 Da	It mimics components of bacterial all walls (mycobacteria) and stimulates an enhanced response. It binds to NOD 2, activates NF-kB	- Effectiveness in treating dangerously lower counts of neutrophil and restoring the number of leukocytes and platelets in cancer patients after radio- and chemotherapy;<br>- Affection the metabolism of certain drugs by modulating the activity of cytochrome PUSO enzymes in liver	- Possible flu-like symptoms, gastrointestinal disturbances and autoimmune reactions	- Neutropenia caused by chemotherapy;<br>- Analgesic effect for animals	Tested in humans and animals	[221]
10	Lycopide	The drugs of microbial origin, Disaccharide-containing muramyl dipeptide<br><br>{NA}	$C_{25}H_{43}N_{5}O_{15}$<br><br>653.27 Da	It activates the anti-infectious protection, cellular and humoral immunity and shows pathogenetic effect from Th-2-type to Th1. It corrects cytopenia by accelerating the process of bone marrow hematopoiesis	- Stimulation of development of both cellular and humoral immune responses and leukopoiesis;<br>- Adjuvant for use in a wide range of vaccines (HIV, DNA) and experiments;<br>- Effectiveness in the treatment of secondary immunodeficiency states	NA	- Chronic recurrent respiratory tract infections, inflammatory diseases of skin and soft tissues, psoriasis, herpetic infections	Tested in humans	[221]

11	Cyclophosphamide (Frindovyx®, Procytox®)	The drugs of microbial origin, Alkylating drugs<br><br>{DB00531}	$C_7H_{15}Cl_2N_2O_2P$<br><br>261.08 Da	It works by 3 different mechanisms: 1) attachment of CH_3 group to DNA synthesis and RNA transcription from the affected DNA; 2) DNA damage via the formation of cross-links and prevents DNA from being separated for synthesis or transcription; 3) the induction of mispairing of the nucleotides leading to mutations	- Effectiveness as immunosuppressive agent for treatment of autoimmune diseases (multiple sclerosis);<br>- Immunosuppressant to prevent transplant rejection and draft-vs-host complications	- Possible hemorrhagic cystitis, amenorrhea, myclosuppression, alopecia and spells of nausea and vomiting reproductive toxicity;<br>- Acute toxic, irritant	- Treatment of certain cancers, lymphomas, myelomas, leukemia, mycosis fungoides, neuroblastoma, ovarian adenocarcinoma, retinoblastoma, and breast carcinoma	Phase IV clinical trials	[222]
12	Sodium nucleinate	The drugs of microbial origin, stimulator leukopoiesis<br><br>{NA}	$C_8H_{10}N_3NaO_4$<br><br>235.06 Da	It regulates the migration of T-lymphocytes and the processes of cooperation of T- and B-lymphocytes, enhances the phagocytic activity of macrophages and the production factors	- Acceleration of tissue regeneration processes;<br>- Stimulation of bone marrow activity and leukopoiesis, immune reactivity in patients with liver disorders	- Possible allergic reactions	- Treatment of secondary immunodeficiency conditions, chronic nonspecific lung diseases, bacterial infections;<br>- Correction of leukopenia and agranulocytosis;<br>- Improvement of the immune status and reduction of signs of immuno-inflammatory syndromes	Tested in humans	[223]
13	Thymosin fraction (TF 5)	The drugs of thymic origin<br><br>{NA}	NA<br><br>MW from 1 000 to 15 000 Da	It stimulates the release of prolactin and growth harmone, β-endorphin and ACTH. It increased T-cell numbers and functions	- Effectiveness to treat cancer, immunodeficiency and infectious diseases	NA	- Stimulation of PRL, $MtTW_{15}$, 7315a cells and GH release, ACTH release	Tested in humans	[224]
14	Thymosin alpha-1 (T-alpha-1) (Zadaxin®)	The drugs of thymic origin<br><br>{NA}	NA<br><br>3 108 Da	It activates TLR-2, TLR-9, DC cells, macrophages, NK cells, cellular and humoral responses and stimulates IL-2, IL-10, IL-12, IFN-α,γ. It inhibits IL-1β, TNF-α and viral replication	- Significantly promotion the proliferation of activated T-lymphocytes;<br>- Protection lymphocytes from immunocytotoxic effects;<br>- Activation the body's T-cell response	- Possible hypersensitivity, nausea, vomiting, neutropenia	- Infection diseases (Hepatitis C, B, sepsis, HIV infection, Pseudomonas, Mold toxicity);<br>- Cancer and chemoprevention;<br>- Immune deficiency diseases;<br>- Attenuation viral replication, IL-1β, TNF-α	Tested in humans	[224]
15	Prothymosin alpha	The drugs of thymic origin<br><br>{NA}	NA<br><br>~ 32 000.0 Da	It has nuclear activity, which shows the ability to generate Tα-1 and Tα-11 by natural processing. It's a cytoplasmic function is releated to the control apoptosome activity counteracting the proapoptotic action of the protein ANP32A, pp32	- Pleiotropic adjuvant activity and immunomodulatory effects (anticancer, antiviral, neuroprotective, cardioprotective);<br>- Unique antineuronal necrosis factor of cortical neurons;<br>- Decreasing the apoptotic response and enhances cell survival;<br>- Reducing the infarct size after myocardial infarction	NA	- Cancer immunotherapy<br>- Potent adjuvant for hepatitis B virus DNA vaccines;<br>- Treatment of infectious diseases (chronic hepatitis B and C, AIDS);<br>- Ischemic stroke	Tested in humans and animals	[225]

16	Beta thymosins T-beta	The drugs of thymic origin<br><br>{NA}	NA<br><br>~ 4 982 Da	It participates in polymerization, cell migration, collagen deposition, wound healing and tissue repair, suppressing TNF-α expression, ferroptosis modulation	- Protection against further damage, reducing programmed cell death, microbial proliferation, inflammation;<br>- Multifaceted effects, neuroprotective properties, anti-inflammatory and potential in regulating miRNA expression	- Variability in individual responses;<br>- Risk of inflammation	- Wound healing<br>- Cardiovascular diseases;<br>- Ocular conditions;<br>- Neurological disorders;<br>- Liver fibrosis;<br>- Anti-inflammatory applications	Tested in humans	[224]
17	Thimulin (Thymulin)	The drugs of thymic origin<br><br>{NA}	$C_{33}H_{54}N_{12}O_{15}$<br><br>858.864 Da	It participates in T-cell differentiation, inflammation and anti-proinflammatory effect, Thymus-Pituitary axis. It activates NK-cells	- Activation of NK-cells;<br>- Influence the expression of phenotypic markers (CD90, CD3, CD4, CD8);<br>-Anti–inflammatory and analgesic properties in the brain;<br>- Strength immune function and support neurological health	- Lack of extensive clinical studies in humans;<br>- Lack of data on long-term safety in humans	- Stimulation of the production of luteinizing hormone;<br>- Increasing the production of GH, PRL, TSH and hormones;<br>- Intra- and extrathymic T-cell differentiation;<br>- Modulating immune response	Never entered clinical trials Tested in rats	[224]
18	Thymic humoral factor	The drugs of thymic origin<br><br>{NA}	NA<br><br>~ 918 Da	It activates HSC and participates in inflammatory cell infiltration and produce cytokines (TNF-α, TGF-β, IL-6) at early stage	- Effectiveness to T-cell responses;<br>- Stimulation of the proliferation of lymphoid cells;<br>- Restoring deficient T-cell function in humans;<br>- Effectiveness to treat cancer or viral diseases	- No toxic or allergic effects observed in treating	- Treatment of hepatitis B, C with α-interferon;<br>- Increasing antiviral activity against cytomegalovirus in animal models;<br>- Controlling liver damage and fibrosis	Tested in humans	[226]
19	Thymopoietin	The drugs of thymic origin<br><br>{NA}	NA<br><br>~ 5 000.0 Da	It differentiates of precursor lymphoid cell	- Significantly increasing of the resistance to infections;<br>- Increasing levels of IL-2 and IFN-γ	- Its regulatory role is complex;<br>- Possible burns and bacterial infections	- Affection in neuromuscular transmission and inducing phenotypic differentiation of T-lymphocyte precursor cells;<br>- Decreasing inflammatory responses;<br>- Support immune homeostasis;<br>- Therapeutic potential	Tested in humans and animals	[224]
20	Thymostimulin	The drugs of thymic origin, the extract from calf thymus<br><br>{NA}	NA<br><br>MW from 1 000 to 12 000.0 Da	It stimulates T-cell proliferation and differentiation. It reduces the incidence of infection in surgery, cancer.	- Restoring T-cell function in immunocompromised patients;<br>- Protection from zidovudine-induced bone marrow toxicity and infection;<br>- Increasing of some immunological parameters	- No adverse reactions, no side effects were observed	- Treatment for cancer and chemotherapy;<br>- Reducing of the risk of death or disease progression;<br>- Increasing $CD4^{+}$, $CD8^{+}$ cell-dependent antibacterial activities	Phase III clinical trials	[224]
21	Thymus factor X (TFX)<br>(TFX-Jelfa®)	The drugs of thymic origin<br><br>{NA}	NA<br><br>Lower than	It stimulates the thymocyte proliferation and then restorates proliferation capabilities	- Its lack of toxicity;<br>- Inhibition of herpes simplex virus type 1 in A 549 cells and proinflammatory cytokine	- NA	- Treatment of subcutaneous or intramuscular injection for patients with primary or secondary	Tested in humans	[227]

			10 000.0 Da	of thymocytes. It weakly inhibits TNF-α, IL-1β and IL-6 by THP-1 monocyte cell line. Also it elevates p38a	production (TNF-α, IL-1β and IL-6) by LPS-stimulated macrophages (THP-1);<br>- Effection on MAPKs		immunodeficiencies and cancer;<br>- For therapeutic immunotropic effects	and animals	
22	Thymomodulin	The drugs of thymic origin, the extract from calf thymus<br><br>{NA}	NA<br><br>MW from 1 000 to 10 000.0 Da	It induces the maturation of T-lymphocytes and effects on T, B, N, K and bone marrow stem cells	- Effectiveness for treating various disease processes, infections, allergies, malignancies;<br>- Improving of the immunological function during the ageing;<br>- Increasing of the leukocyte levels;<br>- Radioprotective effect	- No side effects have been seen in all the patients treated	- Treatment of respiratory infections, asthma, atopic dermatitis, atopic eczema, food allergy, acute hepatitis B, HN, chronic liver diseases	Tested in humans and animals	[227]
23	Thymogen	The drugs of thymic origin<br><br>{NA}	$C_{16}H_{19}N_3O_5$<br><br>333.34 Da	It regulates stem and colony-forming cells and impacts on immunological processes and hematopoiesis. It binds in a human mesenchymal and hematopoietic stem cells to the promoter region of the DNA double helix to lymphocyte cells	- Leading to a more rapid restoration of leukocytes and granulocytes;<br>- Significantly decreasing the case of neutropenia and increasing of leukocytes	- Possible vomiting, nausea, diarrhoea, rash	- Stimulation of the immune response;<br>- Complex therapy of acute and chronic viral, bacterial infections and to correct secondary immunodeficiency during radiation therapy, chemotherapy, antibiotic therapy;<br>- Prevention of suppression of immunity, hemotopoiesis, regeneration processes	Tested in humans	[228]
24	IRX-2	The drugs of thymic origin, protein from rat thymus<br><br>{DB05643}	NA<br><br>~49 000.0 Da	Cytokine-based biologic agent, which stimulates dendritic cells, Natural killer cells and T-lymphocytes	- The safety and feasibility of multi-agent neoadjuvant cytokine immunotherapy	- No significant correlations of immunologic or tumor responses with survival outcomes;<br>- No tumor size changes	- Treatment of cancer and solid tumors;<br>- Promotion of anticancer immune response	Phase II clinical trials	[229]
25	IRX-3	The drugs of thymic origin<br><br>{NA}	NA	Iroquois-class homeodomain protein, it influences to over body weight through the hypothalamus and inhibits beiging and thermogenesis, promoting white adipogenesis and fat storage	- Readily responding of the chondrogenic stimuli;<br>- May be a beneficial during developmental stages to modulate adipogenesis	- Complete absence of IRX-3 in the embryonic fibroblast stage leads to detrimental loss of adipogenic differentiation capacity;<br>- Promotion to obesity	- For the identity, function and adipogenic differentiation of beige adipocyte precursors	Tested in humans and animals	[230]
26	Aldesleukin (Proleukin®)	The drugs of cytokine origin, interleukin drugs<br><br>{DB00041}	$C_{690}H_{1115}N_{177}O_{202}S_6$<br><br>15 314.8 Da	It binds to the IL-2 receptor, which leads to heterodimerization of the cytoplasmic domains of the IL-2R-β and γ and activation of tyrosine kinase JAK3 and phosphorylation of tyrosine residues. It	- Capability of affecting various types of immune cells;<br>- Having immunostimulatory and immunosuppressive effects to treat immune-related diseases	- Possible allergic reaction, capillary leak syndrome, gall bladder disease, hypercalcemia, Crohn's disease, scleroderma;<br>- Toxicity the use of high-dose of drug for treating cancers	- Inducing an adaptive immune response in the treatment of renal all carcinoma;<br>- Kidney and skin cancers	Phase IV clinical trials	[231]

				stimulates growth and differentiation of T-cells				
27	Oprelvekin (Neumega®)	The drugs of cytokine origin, interleukin drugs<br><br>{DB00038}	$C_{854}H_{1411}N_{253}O_{235}S_2$<br><br>19 047.2 Da	It binds to the interleukin 11 receptor which leads to a cascade of signal transduction events. It has thrombopoietic growth factor, which stimulates the proliferation of hematopoietic stem cells and megakaryocyte progenitor cells and induced megakaryocyte maturation resulting in increased platelet production	- Usefulness in longer chemotherapeutic protocols;<br>- Ability to prevent platelet transfusions in multiple cycles of chemotherapy;<br>- The primary treatment of choice for prevention of severe thrombocytopenia	- Function of IL-11 in tissue regeneration;<br>- Negative effect on wound healing	- Stimulation of production of megakaryocytes and platelets in patients with or at risk of thrombocytopenia after chemotherapy	Phase IV clinical trials	[232]
28	Syperlymph (Syperlymph®)	The drugs of cytokine origin, interleukin drugs<br><br>{NA}	NA<br><br>~40 000.0 Da	It stimulates the functional activity of phagocytic cells and activates phagocytosis, cytokine production (IL-1, TNF). It induces antitumor cytotoxicity of macrophages and increases the activity of natural killers	- Contribution of longer preservation of the therapeutic effect of the therapy;<br>- Increasing of duration of the relapse-free period and decreasing of the number of relapses	- Possible allergic reaction and disease	- Comprehensive treatment of herpetic diseases of the urogenital tract, including those complicated by bacterial and other viral infections;<br>- Comprehensive treatment of chronic recurrent uncomplicated cystitis to prolong the period of remission	Tested in humans	[233]
29	Roncoleukin	The drugs of cytokine origin, interleukin drugs<br><br>{NA}	NA	It affects T-lymphocytes, enhancing their proliferation and subsequent synthesis of IL-2. It binds to specific receptors and affects the growth, differentiation and activation of T, B lymphocytes, monocytes, macrophages and langerhans epidermal cells	- Normalization of cytokine balance and antioxidant status;<br>- Decreasing of proinflammatory cytokines	- Hypersensitivity to IL-2 or any component of the drug in the anamnesis	- Treatment of combined immunodeficiency, acute peritonitis, osteomyelitis, sepsis, tuberculosis of the lungs, severe pneumonia, chemical burns, renal cell carcinoma	Tested in humans and animals	[234]
30	Betaleukin (Betaleukin®)	The drugs of cytokine origin, interleukin drugs<br><br>{NA}	NA	It stimulates hematopoiesis and early post-radiation recovery. It accelerates the restoration of stem potential and bone marrow hematopoiesis, granulopoiesis after the damaging effects of cytostatics and ionizing radiation. It increases the inducing differentiation of immunocompetent cell precursors, enhancing lymphocyte proliferation	- Restoration of leukocyte;<br>- Prevention of cell depletion in the bone marrow;<br>- Inhibition of anemia development	- Possible common reactions as chills, headache, fever	- As a leukopoiesis stimulator;<br>- Treatment of toxic leukopenia of II-IV degree, complicating chemo- and radiotherapies of malignant tumors, as a protector of leukopoiesis, secondary immunodeficiency conditions, purulent-septic and purulent-destructive processes, acute emergency total and subtotal effects of ionizing radiation	Tested in humans and animals	[235]

				and increasing antibody formation					
31	Peginterferon alfa-2a (Pegasys®)	The drugs of cytokine origin, interferon drugs {DB00008}	NA 60 000.0 Da	It binds to and activates human type 1 interferon receptors. It also activates JAK/STAT pathway causing them to increase the expression of multiple genes	- An effective therapeutic choice in patients with PV and ET; - Reducing of the Myeloproliferative Neoplasm symptoms and Median JAK-2V6/7 VAF; - Improvement of cell counts, bone marrow cellularity and fibrosis	- Progression of myelofibrosis; - Possible infection diseases, allergic reactions and gastrointestinal disorders	- Treatment of hepatitis B and C infections by activating immune system	Phase IV clinical trials	[236]
32	Peginterferon alfa-2b (Pegintron®, Sylatron®)	The drugs of cytokine origin, interferon drugs {DB00022}	NA 31 000.0 Da	It binds to and activates human type 1 interferon receptors causing them to dimerize. This activates the JAK/STAT pathway. It also activates the nuclear factor kB pathway	- Being safest and well-trolerated drug, efficacious in patients with PV and ET	- Possible influenza-like sumptoms, hyperglobulinemia, elevated alanine aminotransferase, subclinical hypothyroidism; - Progression of myelofibrosis	- Stimulation the innate antiviral response in the treatment of hepatitis B and C, genital warts and some cancers	Phase IV clinical trials	[237]
33	Ropeginterferon alfa-2b (Besremi®)	The drugs of cytokine origin, interferon drugs {DB15119}	$C_{16}H_{29}N_3O_6(C_2H_4O)n (C_2H_4O)n$ ~60 000.0 Da	It binds to the IFNAR and activates downstream JAK/STAT signaling. It increases T-cell, macrophage and natural killer cells	- A strong anti-neoplastic effect at a higher mitiating-dose; - Effectiveness depleting JAK2V617F-carrying neoplastic cells in patients with PV; - No disease progression	- Possible cause of renal and urinary disorders (Proteinuria), Hyperuricalmia and Hypertriglyceri-daemia	- Treatment of a specific form of blood cancer	Phase IV clinical trials	[238]
34	Interferon gamma	The drugs of cytokine origin, interferon drugs {DB15753}	$C_{761}H_{1206}N_{214}O_{225}S_6$ 17 145.65 Da	It signals as an antiparallel homodimeric glycoprotein, acting at the complex of IFNGR1 and IFNGR2 after direct binding to IFNGR1. It activates the JAK/STAT, MAPK, PI3-kinase pathways. It also activates macrophages and regulates Th1/Th2 balance and controls cellular proliferation and apoptosis	- Treatment is helpful for preventing bladder cancer recurrence	- Any improvement in clinical parameters in melanoma, soft tissue sarcoma and breast cancer	- Treatment of chronic granulamatous disease, osteopetrosis, idopathic pulmonary fibrosis	Phase III clinical trials	[239]
35	Levamisole (Ergamisol Tab®)	The drugs of chemically pure origin {DB00848}	$C_{11}H_{12}N_2S$ 204.291 Da	It stimulates the formation of antibodies to various antigens, enhance T-cell response by stimulating T-cell activation and proliferation, potentiate monocyte and macrophage functions including phagocytosis,	- Short course of Lev achieves a significant, transient reduction in loa MFD	- Possible an allergic reaction, blood problems, nervous system problems and decreasing of bone marrow function	- Treatment of parasitic, viral and bacterial infections and various immunemediated diseases and cancers, Rheumatoid arthritis, vitiligo	Phase IV clinical trials	[240]

				chemotaxis, neutrophil mobility, adherence					
36	Diucifon	The drugs of chemically pure origin<br><br>{NA}	$C_{22}H_{20}N_6O_{10}S_3$<br><br>624.623 Da	It helps to eliminate the deficiency of cellular and humoral components of immunity, stimulates RNA synthesis and significantly reduces the level of circulating immune complexes in patients with predominantly articular rheumatoid arthritis	- Stimulating effect on the cellular component of the immune system;<br>- Maintaining remission in children with clinical forms of bronchial asthma;<br>- Exacerbation and addition of acute respiratory infections and other infectious diseases	- Possible general weakness, decreasing appetite, dyspeptic symptoms, toxic hepatitis, hypochromic anemia	- Treatment of leprosy, immunodeficiency state of body, including dermatoses, rheumatoid arthritis, tuberculosis, chronic nonspecific lung diseases	Tested in humans	[241]
37	Dibazol (Bendazole®, Dibazil®)	The drugs of chemically pure origin<br><br>{NA}	$C_{14}H_{12}N_2$<br><br>208.265 Da	It increases the activity of natural killer cells and promoting the production of interferons, proteins. It stimulates the central nervous system, which may contribute to its vasodilatory effects	- Stimulation of the intracellular production of DNA and protein;<br>- Improvement of health condition and physical work capacity;<br>- Acceleration of fibrinolysis	- Possible gastrointestinal disturbances (nausea, vomiting, diarrhea)	- Treatment of arterial hypertension, hypertensive crisis, spasms of smooth muscles of internal organs	Tested in humans	[242]
38	Bemitil	The drugs of chemically pure origin<br><br>{NA}	$C_9H_{10}N_2S$<br><br>624.623 Da	It activates protein synthesis (RNA), cell genome, protein enzymes (in particular, enzymes of glunoneogenesis and mitochondrial oxidation) resulting this increased energy production in the cell and antioxidant protection	- Antioxidant potential	- The challenge of identifying its urinary metabolites	- Complex therapy of functional astheria	Tested in humans and animals	[243]
39	Ionic Trimecaine derivatives	The drugs of chemically pure origin<br><br>{NA}	$C_{16}H_{27}N_2O$<br>$C_{17}H_{29}N_2O$<br>$C_{18}H_{31}N_2O$<br><br>263.2-291.2 Da	NA	- Stimulation of leukopoiesis, platelet- and erythropoiesis	- NA	- Can be a stimulator of leukopoiesis	Tested in animals	[244]
40	Mavorixafor (Xolremdi®)	The drugs of chemically pure origin<br><br>{DB05501}	$C_{21}H_{27}N_5$<br><br>349.48 Da	It regulates immune cell trafficking and homeostasis and activates G-protein signaling, calcium mobilization and ERK/AKT activation. It binds to the receptor and promotes the trafficking and homing of leukocytes to and from the bone marrow compartment	- Reducing infection frequency, severity, duration and antibiotic use;<br>- Increasing in LS mean TAT_{ANC} and TAT_{ALC}	- No related serious TEAEs were observed	- Treatment of infections trichomoniasis, amebiasis, giardiasis	Tested in humans	[245]
41	Ornidazole	The drugs of chemically pure origin	$C_7H_{10}ClN_3O_3$	It acts as an electron acceptor in the metabolism of the bacteria and causes	- Improvement the clinical efficacy;	- Some deficiencies in the bling method of elinical research	- Treatment of infections trichomoniasis, amebiasis, giardiasis	Tested in humans	[246]

		{DB13026}	219.63 Da	growth disturbances in the susceptible microorganism	- Reducing of the duration of pain and the density of oral bacteria				
42	Tetramisole	The drugs of chemically pure origin {DB16561}	$C_{11}H_{12}N_2S$ 204.29 Da	It causes paralysis by blocking the neuromuscular transmission, leading to the expulsion of the parasites from the digestive tract	- Selectively increasing of the I_{k1} current in ARVMs; - Improvement iso-induced cardiac remodeling *in vivo*; - High selective affinity with Kir2.1	- Limited efficacy on I_{k1} and AP	- Treatment of a wide variety of dermatophyte infections and candidiasis	Tested in humans	[247]
43	Clotrimazole (Alevazol®, Dermacinrx®, Therazole Pak®, Lotriderm®, Lotrimin®, Lotrimin AF®, Lotrisone®, Mycelex®)	The drugs of chemically pure origin {DB00257}	$C_{22}H_{17}ClN_2$ 344.837 Da	It causes inhibition of ergosterol biosynthesis, an essential constituent of fungal cell membranes. It shows inhibition of sarcoplasmic reticulium Ca^{2+} ATPase, depletion of intracellular calcium and blocking of calcium-dependent potassium channels and voltage-dependent calcium channels	- Effectiveness to treat of otomycosis	- Possible erythema, stinging, burning and general irritation of the skin and cramps	- Treatment of a wide variety of dermatophyte infections and candidiasis	Phase IV clinical trials	[248]
44	Metronidazole (Flagyl®, Flagystatin®, Likmez®, Metrocream®, Metrogel®, Metrolotion®, Nidagel®, Noritate®, Nuvessa®, Pylera®, Rosadan®, Vandazole®)	The drugs of chemically pure origin {DB00916}	$C_6H_9N_3O_3$ 171.154 Da	It binds to deoxyribonucleic acid and electron-transport proteins of organisms, blocks nucleic acid synthesis. It enters cells by passive diffusion	- Improvements in clinical, radiographic and microbiological parameters (reducing PPD, increasing CAL and radiographic bone, decreasing counts porphyromonas gingivalis, Tannerella forsythia and Campylobacter rectus)	- Possible peripheral neuropathy, central nervous system toxicity, disulfiram-like effect	- Treatment of trichomoniasis, amebiasis, inflammatory lesions of rosacea and bacterial infections and prevent postoperative infections	Phase IV clinical trials	[249]
45	Flutrimazole	The drugs of chemically pure origin {DB13425}	$C_{22}H_{16}F_2N_2$ 346.381 Da	NA	- Drastic antifungal effect	- Limited solubility in water may cause of its low bioavailability	- Treatment of pityriasis capitis and seborrheic dermatitis	NA	[250]
46	Isoconazole	The drugs of chemically pure origin {DB08943}	$C_{18}H_{14}Cl_4N_2O$ 416.129 Da	It inhibits the steroid 17-α-hydroxylase/17,20 lyase	- Effectiveness to treat the otomycosis	- NA	- Treatment of fungal infections	Tested in humans	[251]
47	Miconazole (Aloe Vesta Antifungal®, Baza®, Critic-aid Clear®, Desenex®, Fungoid®, Inzo®, Lagicam®, Lotrimin AF®,	The drugs of chemically pure origin {DB01110}	$C_{18}H_{14}Cl_4N_2O$ 416.129 Da	It inhibits the CYP450-14-α-lanosterol demethylase enzyme. It also inhibits fungal peroxidase and catalase while not affecting NADH oxidase activity, leading to increased	- Effectiveness to treat the oral candidiasis symptoms; - Reducing candida colonization; - Significant reduction in the number of colonies forming units of Candida albicans	- No adverse reactions	- Treatment of a variety fungal infections	Phase IV clinical trials	[252]

	Micatin®, Micro-guard®, Monistat®)			production of reactive oxygen species. It causes a rise in intracellular levels of farnesol					
48	Morinidazole	The drugs of chemically pure origin {DB15098}	$C_{11}H_{18}N_4O_4$ 270.289 Da	It diffuses into the organism, inhibits protein synthesis with DNA and causes a loss of helical DNA structure and strand breakage	- Safety and effectiveness to treat pelvic inflammatory disease	- No adverse reactions	- Treatment of worm infection in humans	Phase IV clinical trials	[253]
49	Flubendazole	The drugs of chemically pure origin {DB08974}	$C_{16}H_{12}FN_3O_3$ 313.283 Da	It works by interacting with tubulin, which disrupts the microtubule framework and interferes with the movement of secretory vesicles in worm absorptive tissues	- Good oral bioavailability	- Flubendazole-induced changes in haematological, lymphoid and gastrointestinal systems	- Treatment of worm infection	Tested in animals	[254]

Abbreviations: ACTH - Adrenocorticotropic hormone, AEs - adverse events, AIDS - acquired immunodeficiency syndrome, AKT - protein kinase B, ANP32A - Acidic leucine rich nuclear phosphoprotein-32A, ARDS - acute respiratory distress syndrome, ARVMs - adult rat ventricular myocytes, ATPase – adenylpyrophosphatase, BM-MSCs- Bone marrow mesenchymal stem cells, CAL - clinical attachment level, CD - cluster of differentiation, CIN - chemotherapy-induced neutropenia, c-KIT – CD117 tyrosine kinase receptor, CYP450 - cytochrome P450, DNA - deoxyribonucleic acid, EPK - eukaryotic protein kinase, ERK - extracellular signal-regulated kinase, G-CSF - granulocyte colony-stimulating factor, GH - growth hormone, GI – gastrointestinal, GM-CSF-R-alpha, GVHD - Graft-versus-host disease, HIV - human immunodeficiency viruses, HN - hemagglutinin-neuraminidase, HSC - hepatic stellate cells, IFNAR - interferon-alpha/beta receptor, IFNGR1- interferon gamma receptor 1, IFN-α,γ - interferon-alpha, gamma, IL – interleukin, IL-1β - interleukin-1 beta, irAEs - immune-related adverse events, IRX - iroquois homeobox, JAK - Janus kinase, Kir2.1 - inwardly rectifying potassium, MAPKs - mitogen-activated protein kinases, MFD - mutation frequency decline, $MtTW_{15}$ - rat pituitary tumors, NF-kB - nuclear factor kappa-light-chain enhancer of B cells, NK-cells – natural killer cells, NOD - nucleotide-binding oligomerization domain, PI3-kinase - phosphoinositide 3-kinase, p38a - mitogen-activated protein kinases, pp32 - protein phosphatase 32, PPD - purified protein derivative, PRL – prolactin, PUSO enzymes, RNA - ribonucleic acid, SR-aGVHD - steroid refractory of graft-versus-host disease, STAT - signal transducer/activator of transcription, TAT_{ALC} - threshold absolute lymphocyte count, TAT_{ANC} - threshold absolute neutrophil count, TEAEs - treatment-emergent adverse events, TGF-β - Transforming growth factor beta, Th - T helper, THP-1- human leukemia monocytic cell line, TLR - Toll-like receptor, T-lymphocytes - white blood cell, TNF-α - tumor necrosis factor-alpha, TSH - thyroid stimulating hormone, Tα – thymosin alpha, VAF - variant allele frequency.

References:

213. Kurtzberg, J.; Burke, E.; Hayes, J.; Rose, E.; Itescu, S. Ryoncil (Remestemcel-L) for third-line treatment of SR-aGVHD in adolescents and adults. *Transplant. Cell. Ther. ASTCT*. **2025**, *31*, S288 - S289. https://doi.org/10.1016/j.jtct.2025.01.439
214. Drugs and Lactation Database (LactMed®). Available online: https://www.ncbi.nlm.nih.gov/books/NBK501373/ (accessed on 15 Oct 2024).
215. Borouchaki, A.; Roquetaillade, C.; Barthélémy, R.; Mebazaa, A.; Chousterman, B.G. Immunotherapy to treat sepsis induced-immunosuppression: Immune eligibility or outcome criteria, a systematic review. *J. Crit. Care.* **2022**, *72*. https://doi.org/10.1016/j.jcrc.2022.154137
216. Yokoe, T.; Yoshinami, T.; Nozawa, K.; Ozaki, Y.; Nishio, H.; Tsuchihashi, K.; Ichihara, E., Miura, Y.; Endo, M.; Yano, S.; Maruyama, D.; Susumu, N.; Takekuma, M.; Motohashi, T.; Ito, M.; Baba, E.; Ochi, N.; Kubo, T.; Uchino, K.; Kimura, T.; Kamiyama, Y.; Nakao, S.; Tamura, S.; Nishimoto, H.; Kato, Y.; Sato, A.; Takano, T. Efficacy and safety of dose-dense chemotherapy for early-stage breast cancer under prophylactic pegfilgrastim administration: a systematic review and meta-analysis from clinical practice guidelines for the use of G-CSF 2022. *Int. J. Clin. Oncol.* **2025**, *30*, 674-683. https://doi.org/10.1007/s10147-025-02716-2
217. Khan, S.Z. Eflapegrastim (Rolvedon) for prevention of chemotherapy-induced febrile neutropenia. *Med. Lett. Drugs Ther*. **2023**, *65*, 83-84. https://doi.org/10.58347/tml.2023.1677b .

218. Glaspy, J.A.; Bondarenko, I.; Tjulandin, S.; Auerbach, M. Efbemalenograstim alfa, a long-acting granulocyte colony-stimulating factor fusion protein without pegylation, versus pegfilgrastim for management of chemotherapy-induced neutropenia in patients with breast cancer: results of a phase III randomized noninferiority trial. *JCO oncol. adv.* **2025**, *2* https://doi.org/10.1200/OA-24-00074
219. Baldo, B.A. *Cytokines. Safety of Biologics Ther.* **2016**, 217–261. https://doi.org/10.1007/978-3-319-30472-4_5
220. Dougan, M; Nguyen, L.H.; Buchbinder, E.I.; Lazarus, H.M. Sargramostim for prophylactic management of gastrointestinal immune-related adverse events of immune checkpoint inhibitor therapy for cancer. *Cancers (Basel).* **2024**, *16*, 501. https://doi.org/10.3390/cancers16030501
221. Guryanova, S.V.; Khaitov, R.M. Strategies for using muramyl peptides - modulators of innate immunity of bacterial origin - in medicine. *Front. Immunol*. **2021**, *12*, 607178. https://doi.org/10.3389/fimmu.2021.607178
222. Cyclophosphamide. Available online: https://www.ncbi.nlm.nih.gov/books/NBK553087/ (accessed on 03 Jul 2023).
223. Shved, M.; Prokopovych, O.; Lypovetska, S.; Heryak, S.; Kitsak, Y. Efficiency of bioflavonoid quercetin and rna-containing drug sodium nucleinat in complex treatment of patients with myocardial infarction and functional liver disorders. *Health Probl. Civiliz.* **2017**, *11*, 293-299. https://doi.org/10.5114/hpc.2017.71891
224. Dominari, A.; Hathaway III, D.; Pandav, K.; Matos, W.; Biswas, S.; Reddy, G.; Thevuthasan, S.; Khan, M.A.; Mathew, A.; Makkar, S.S.; Zaidi, M.; Fahem, M.M.M.; Beas, R.; Castaneda, V.; Paul, T.; Halpern, J.; Baralt, D. Thymosin alpha 1: A comprehensive review of the literature. *World J. Virol.* **2020**, *9*, 67-78. https://doi.org/10.5501/wjv.v9.i5.67
225. Samara, P.; Ioannou, K.; Tsitsilonis, O.E. Prothymosin alpha and immune responses: Are we close to potential clinical applications? *Vitam Horm.* **2016**, *102*, 179-207. https://doi.org/10.1016/bs.vh.2016.04.008
226. Lunin, S.; Khrenov, M.; Glushkova, O.; Parfenyuk, S.; Novoselova, T.; Novoselova, E. Precursors of thymic peptides as stress sensors. *Expert Opin. Biol. Ther.* **2020**, *20*, 1461–1475. https://doi.org/10.1080/14712598.2020.1800636
227. Zimecki, M.; Kochanowska, I.E.; Zaczyńska, E.; Kocięba, M.; Artym, J.; Zambrowicz, A.; Matwiejczyk, M.; Besman, M.; Kuchar, K.; Skotnicki, A. Immunoregulatory actions of calf thymus extract (TFX®) in vitro in relation to its effect on expression of mitogen activated protein kinases. *Int. Immunopharmacol*. **2023**, *118*. https://doi.org/10.1016/j.intimp.2023.109995
228. Deigin, V.; Linkova, N.; Vinogradova, J.; Vinogradov, D.; Polyakova, V.; Medvedev, D.; Krasichkov, A.; Volpina, O. The first reciprocal activities of chiral peptide pharmaceuticals: Thymogen and thymodepressin, as examples. *Intern. J. Mol. Sci.* **2024**, *25*, 5042. https://doi.org/10.3390/ijms25095042
229. Wolf, G.T.; Bellile, E.; Mauquoi, C.; Nguyen, A.; Sartor, M.; Liu, S.; Rozek, L.; McHugh, J.B. Neoadjuvant cytokine (IRX-2) immunotherapy for resectable oral cavity carcinoma: Final results of the INSPIRE trial. *OOR*. **2025**, *13*. https://doi.org/10.1016/j.oor.2024.100706
230. Bjune, J.; Lawrence-Archer, L.; Røsland, G.V.; Tronstad, K.J.; Njølstad, P.R.; Sagen, J. V.; Dankel, S.N.; Mellgren, G. The homeobox factor IRX-3 maintains adipogenic identity. Metabolism. 2020, 103. https://doi.org/10.1016/j.metabol.2019.154014
231. Roser, L.A.; Sommer, C.; Iannazzo, S.O.; Sakellariou, C.; Waibler, Z.; Gogesch, P. Revival of recombinant IL-2 therapy – approaches from the past until today. *J. Immunotoxicol.* **2024**, *21*, 38-47. https://doi.org/10.1080/1547691X.2024.2335219
232. Soriano, R.A.; Gonzalez, M. Evaluating the effect of oprelvekin on cardiac repolarization in subjects with chemotherapy-induced thrombocytopenia: An observational chart review of a phase 2 clinical trial in Laredo, Texas. *JCRS*. **2017**, *5*. https://www.annexpublishers.com/articles/JCRS/5204-Evaluating-the-Effect-of-Oprelvekin-on-Cardiac-Repolarization-in-Subjects-with-Chemotherapy-Induced-Thrombocytopenia.pdf
233. Gyaurgiev, T.A.; Kuzmenko, A.V.; Kuzmenko, V.V.; Zolotukhin, O.V.; Madykin, Yu.Y.; Avdeev, A.I. Evaluation of the effectiveness of various schemes of using the immunomodulatory drug Superlimf® in the prevention of relapses of chronic abacterial prostatitis (in Russ). *Urology*. **2024**, *1*, 71-79. https://dx.doi.org/10.18565/urology.2024.1.71-79

234. Giesinger, O.A.; Laknitskaya, A.O.; Ziganshin, O.R. Dynamics of antioxidant protection factors, cytokines in patients with chronic streptoderma, correction methods (in Russ). *Russ. J. Immunol.* **2021**, *24*, 337-342. https://doi.org/10.46235/1028-7221-984-DOA
235. Sycheva, L.P.; Rozhdestvenskii, L.M. The role of immunomodulator betaleukin in recovery of hepatocytic ploidy profile in delayed terms after irradiation. *Bull. Exp. Biol. Med.* **2020**, *169*, 463–466. https://doi.org/10.1007/s10517-020-04909-3
236. Sørensen, A.L.; Skov, V.; Kjær, L.; Bjørn, M.E.; Eickhardt-Dalbøge, C.S.; Larsen, M.K.; Nielsen, C.H.; Thomsen, C.; Gjerdrum, L.M.R.; Knudsen, T.A.; Ellervik, C.; Overgaard, U.M.; Andersen, C.L.; Hasselbalch, H. Combination therapy with ruxolitinib and pegylated interferon alfa-2a in newly diagnosed patients with polycythemia vera. *Blood Adv.* **2024**, *8*, 5416–5425. https://doi.org/10.1182/bloodadvances.2024013170
237. Chang, L.; Cai, H.; Cao, X.; Li, J.; Zhou, D.; Duan, M. Efficacy and safety of peginterferon alfa-2b in 95 patients with myeloproliferative neoplasms:a single center retrospective analysis. *Blood.* **2022**, *140*, 12244. https://doi.org/10.1182/blood-2022-168360
238. Suo, S.; Fu, R.F.; Qin, A.; Shao, Z.; Bai, J.; Zhou, H.; Xu, N.; Chen, S.; Zuo, X.; Du, X.; Duan, M.; Wang, L.; Li, P.; Zhang, X.; Zhang, S.; Wu, D.; Zhang, J.; Xiao, Z.; Zhang, L.; Jin, J. Molecular remission uncoupled with complete haematological response in polycythaemia vera treatment with ropeginterferon alfa-2b. *Br. J. Haematol*. **2024**, *205*, 2510-2514. https://doi.org/10.1111/bjh.19846
239. Singh, S.; Chakrabarti, R. Challenges of using IFNγ in clinical settings. *Cancer Res.* **2023**, *83*, 2093-2095. https://doi.org/10.1158/0008-5472
240. Chesnais, C.B.; Hemilembolo, M.C.; Sahm, B.A.; Toutin, F.; Djeutassong, E.; Nga-Elomo, N.; Cuer, B.; Ntsiba-N'Goulou, M.A.; Pakat, M.; Pion, S.D.S.; Missamou, F.; Boussinesq, M.; Campillo, J.T. Safety and efficacy of 3- and 5-day regimens of levamisole in loiasis: a randomized, placebo-controlled, double-blind clinical trial. *Nat. Commun.* **2025**, *16*, 6191. https://doi.org/10.1038/s41467-025-61479-6
241. Anokhina, A.V.; Silantyeva, E.N.; Ruvinskaya, G.R. Clinical case of isolated lesion of oral mucosa by dermatitis herpetiformis. *Bio. Nano. Sci.* **2020**, *10*, 311–314. https://doi.org/10.1007/s12668-019-00699-9
242. Axmedov, S.J.; Ergashov, B.K. Immunomodulatory function of dibazol drug. *Education science and innovative ideas in the world*. **2024**, *38*, 83–87. https://newjournal.org/01/article/view/11231/10883
243. Belinskaia, D.A.; Savelieva, E.I.; Karakashev, G.V.; Orlova, O.I.; Leninskii, M.A.; Khlebnikova, N.S.; Shestakova, N.N.; Kiskina, A.R. Investigation of bemethyl biotransformation pathways by combination of LC-MS/HRMS and in silico methods. *Int. J. Mol. Sci.* **2021**, *22*, 9021. https://doi.org/10.3390/ijms22169021
244. Baktybayeva, L.; Yu, V.; Zazybin, A.; Zolotareva, D.; Dauletbakov, A. Activation of leukopoiesis in rat blood with trimecaine-based ionic compounds. *Biomed. Res. Int.* **2020**, *7636290.* https://doi.org/10.1155/2020/7636290
245. Badolato, R.; Alsina, L.; Azar, A.; Bertrand, Y.; Bolyard, A.A.; Dale, D.; Deyà-Martínez, À.; Dickerson, K.E.; Ezra, N.; Hasle, H.; Kang, H.J.; Kiani-Alikhan, S.; Kuijpers, T.W.; Kulagin, A.; Langguth, D.; Levin, C.; Neth, O.; Olbrich, P.; Peake, J.; Rodina, Y.; Rutten, C.E.; Shcherbina, A.; Tarrant, T.K.; Vossen, M.G.; Wysocki, C.A.; Belschner, A.; Bridger, G.J.; Chen, K.; Dubuc, S.; Hu, Y.; Jiang, H.; Li, S.; MacLeod, R.; Stewart, M.; Taveras, A.G.; Yan, T.; Donadieu, J. A phase 3 randomized trial of mavorixafor, a CXCR4 antagonist, for WHIM syndrome. *Blood.* **2024**, *144*, 35-45. https://doi.org/10.1182/blood.2023022658
246. Du, R.; Ba, K.; Yang, Y.; Zhao, Y.; Lin, Y. Efficacy of ornidazole for pericoronitis: a meta-analysis and systematic review. *Arch. Med. Sci.* **2024**, *20*, 189-195. https://doi.org/10.5114/aoms/171907
247. Liu, Q.; Sun, J.; Dong, Y.; Li, P.; Wang, J.; Wang, Y.; Xu, Y.; Tian, X.; Wu, B.; He, P.; Yu, Q.; Lu, X.; Cao, J. Tetramisole is a new IK1 channel agonist and exerts IK1-dependent cardioprotective effects in rats. Pharmacol. Res. Perspect. 2022, 10. https://doi.org/10.1002/prp2.992
248. Ansley, J.F.; Bernal-Sprekelsen, M.; Butehorn, H.F.; Todorov, S., Tzvetkov, V.; Douglis, F.; Georgiev, K.; Moreira da Silva, F. Pooled analysis of 2 randomized clinical trials to evaluate the efficacy and safety of clotrimazole 1% otic solution for the treatment of otomycosis in adults. *J. Otolaryngol. Head & Neck Surgery.* **2025**, 54. https://doi.org/10.1177/19160216251330629
249. Blanco, C.; Pico, A.; Dopico, J.; Gándara, P.; Blanco, J.; Liñares, A. Adjunctive benefits of systemic metronidazole on non-surgical treatment of peri-implantitis. A randomized placebo-controlled clinical trial. *J. Clin. Periodontol*. **2022**, *49*, 15-27. https://doi.org/10.1111/jcpe.13564

250. Reddy, M.R.; Patnaik, S.S. Design and in vitro characterization of flutrimazole microspheres loaded topical emulgel. *Asian J. Pharm. Clin. Res.* **2019**, *12*, 242–251. https://doi.org/10.22159/ajpcr.2019.v12i9.34341
251. Gülüstan, F.; Abakay, M.A.; Demir, E. Efficacy of topical isoconazole nitrate in the treatment of otomycosis. *Am. J. Otolaryngol.* **2021**, *42*, 102961. https://doi.org/10.1016/j.amjoto.2021.102961
252. Gamil, Y.; Hamed, M.G.; Elsayed, M.; Essawy, A.; Medhat, S.; Zayed, S.O.; Ismail, R.M. The anti-fungal effect of miconazole and miconazole-loaded chitosan nanoparticles gels in diabetic patients with Oral candidiasis-randomized control clinical trial and microbiological analysis. *BMC Oral Health.* **2024**, *24*, 196. https://doi.org/10.1186/s12903-024-03952-0
253. Zhou, T.; Yuan, M.; Cui, P.; Li, J.; Jia, F.; Wang, S.; Liu, R.. Effectiveness and safety of morinidazole in the treatment of pelvic inflammatory disease: A multicenter, prospective, open-label phase IV trial. *Front Med (Lausanne).* **2022**, *9*, 888186. https://doi.org/10.3389/fmed.2022.888186
254. Lachau-Durand, S.; Lammens, L.; van der Leede, B.J.; Van Gompel, J.; Bailey, G.; Engelen, M.; Lampo, A. Preclinical toxicity and pharmacokinetics of a new orally bioavailable flubendazole formulation and the impact for clinical trials and risk/benefit to patients. *PLoS Negl Trop Dis.* **2019**, *13*, e0007026. https://doi.org/10.1371/journal.pntd.0007026
